# Profiling the Main Cell Wall Polysaccharides of the Cultivated Eggplant (*Solanum melongena* L.) Using CoMPP and ELISA

**DOI:** 10.3390/plants15203195

**Published:** 2026-10-18

**Authors:** Eugene Badenhorst, Laura Toppino, Paul Hills, Bodil Jørgensen, Giuseppe Leonardo Rotino, Ignacio Ezquer, John P. Moore

**Affiliations:** 1 South African Grape and Wine Research Institute, Department of Viticulture and Oenology, Faculty of AgriSciences, Stellenbosch University, Stellenbosch 7602, South Africa; ebaden@sun.ac.za; 2 CREA Research Centre for Genomics and Bioinformatics, Via Paullese 28, 26836 Montanaso Lombardo, Italy; laura.toppino@crea.gov.it (L.T.); giuseppeleonardo.rotino@crea.gov.it (G.L.R.); 3 Institute for Plant Biotechnology, Department of Genetics, Faculty of AgriSciences, Stellenbosch University, Stellenbosch 7602, South Africa; phills@sun.ac.za; 4 Department of Plant and Environmental Sciences, University of Copenhagen, 1871 Copenhagen, Denmark; boj@plen.ku.dk; 5 Dipartimento di Bioscienze, Università degli Studi di Milano, 20122 Milano, Italy; juan.ezquer@unimi.it

**Keywords:** eggplant, polysaccharides, glycoproteins, plant cell wall polymers, ELISA, CoMPP, alcohol-insoluble residue, VizELISA

## Abstract

The eggplant (*Solanum melongena* L.) is a commercially important fruit crop; however, research on the cell wall polysaccharide composition is limited. The aim of this study was to provide a plant cell wall profile for eggplant for future research and breeding efforts. A set of cell wall-directed probes was used on three representative eggplant varieties: two genetic lines, 305E40 and 67/3, as well as one commercial cultivar, Black Beauty. Leaf, stem, and root material was analyzed in the case of 305E40 and 67/3, while fruit (skin and flesh) and leaves (at five stages of development) were analyzed in the case of Black Beauty. The results confirmed the presence of a broad range of pectin and hemicellulose epitopes. The presence of AGP and extensin epitopes in eggplant tissues was also confirmed. An ELISA, automated data processing, CoMPP and GC-MS for eggplant cell wall characterization provide a basis for analyzing other commercially important crops to identify breeding targets. These methods are powerful tools for monitoring postharvest fruit quality “texture” and studying plant stress responses.

## 1. Introduction

The eggplant (*Solanum melongena* L.) (also referred to as an aubergine or brinjal) is a commercially important fruit crop and is culinarily considered a vegetable. Research on eggplants has included, among other topics, sequencing the genome [1,2,3] as well as analyzing the polyphenolics (especially anthocyanins in the skin and phenolic acids in the flesh) and nutritional content of eggplant fruits [4,5,6,7]. Eggplant cell walls have yet to be characterized extensively in terms of polysaccharide and glycoprotein composition. Most research that has been performed specifically on eggplant cell wall (CW) polysaccharides has focused on fruit, especially in the context of postharvest cold storage and treatments to prevent chilling injuries in eggplant fruit [8,9,10].

A study by Cornuault et al. [11] showed that high LM25 (xylosyl/galactosyl residues of xyloglucan) signals were observed relative to LM15 (xylosyl residues of xyloglucan) signals in tomato and eggplant, which may be a consequence of the unique arabinoxyloglucan found in the cell walls of the *Solanaceae* [12]. The rhamnogalacturonan-I (RG-1) sidechain: LM5 (galactan) was shown to be predominant in eggplant. Furthermore, eggplant produced signals for both xylan probes tested, LM11 and LM28. This study by Cornuault et al. [11] is one of the main contributors to our knowledge of the composition of eggplant fruit cell walls; however, the study was limited in that only 11 probes (all specific to pectic and hemicellulosic compounds) were used, with no probes for cellulose or glycoproteins such as extensins or arabinogalactan proteins (AGPs). Additionally, only the fruit tissue from a single genotype was included, which was homogenized with no separation of fruit skin from flesh.

Other eggplant cell wall research has mainly focused on the effect of cold storage on eggplant cell walls, especially on pectic polysaccharides. A study on eggplant fruit (cultivar: Y-99 Sulun F1) by Alkan et al. [8] investigated the effects of different postharvest treatments on the activity of cell wall-modifying enzymes (pectin methylesterase, polygalacturonase, α-galactosidase, β-galactosidase, β-1,4-glucanase) as well as their impact on fruit firmness and shelf life. They found that fruits treated with 1-MCP (1-methylcyclopropene) and CaCl_2_ had improved cold storage shelf life and improved quality, while cold storage of untreated fruits saw rapid degradation and a loss in fruit quality. This is likely because eggplants are prone to chilling injuries when subjected to temperatures below 10 °C [13]. Another study looked at the effects of pectin methylesterase, polygalacturonase, β-galactosidase and cellulase on pectin in the context of heat treatment of subsequently cold-stored eggplant fruits [14]. Heat treatment effectively prolonged the shelf-life of eggplant fruits by delaying the activity of these cell wall-modifying enzymes. Another study showed that melatonin treatment can delay fruit softening and senescence [10]. Fruits treated with melatonin showed reduced expression of genes for pectin methylesterase, polygalacturonase and cellulase, as well as genes related to senescence. In a study by Banjongsinsiri et al. [9], the mAbs JIM5 and JIM7 were used to visualize how fruits infused with pectin methylesterase and CaCl_2_ showed varying degrees of pectin methyl-esterification based on the treatment received. In terms of hemicellulose, Kato et al. [12] highlighted how the *Solanaceae* and *Gramineae* contain xyloglucan featuring more unsubstituted glucose residues when compared to most other dicotyledonous plants. In addition to fruits, to our knowledge, other eggplant organs have not been the focus of extensive cell wall research.

The purpose of this study was to characterize the major classes of polysaccharides and glycoproteins present in eggplant cell walls across different organs. Additionally, we anticipate that these baseline data can be used to identify biomarkers linked to quality (e.g., texture) and other fruit physiological parameters (e.g., firmness).

## 2. Results

This study aimed to characterize the eggplant cell wall using ELISA, CoMPP, and GC-MS [15,16,17,18,19] for three eggplant genotypes, namely, the commercial cultivar “Black Beauty” and the lines “305E40” and “67/3”. This study provides an in-depth analysis of eggplant cell walls and confirms the presence of several plant cell wall polymer (PCWP)-related epitopes in eggplant tissues. The parental lines (“305E40” and “67/3”) of a recombinant inbred population (RIL) [15] and the commercial variety “Black Beauty” were selected for the purposes of our analysis because of the availability of genomic datasets and commercial relevance.

### 2.1. Monosaccharide Identification and Quantification by GC-MS

**Figure 1:**
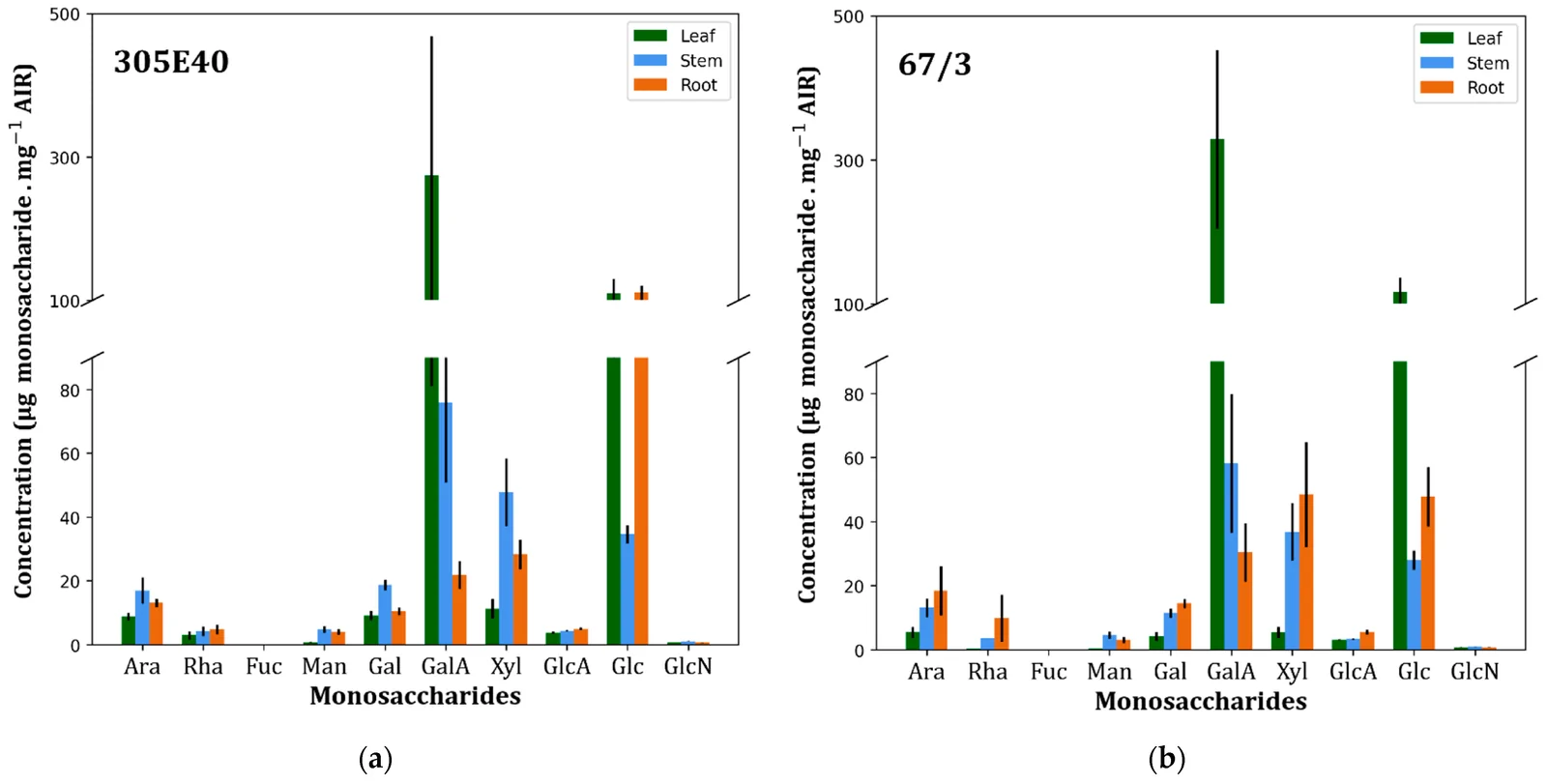
Cell wall monosaccharide composition of the eggplant varieties 305E40 and 67/3 across three tissue types (leaf, stem, and root). The concentration of each monosaccharide is expressed as µg monosaccharide per mg alcohol-insoluble residue (AIR). Gas chromatography–mass spectrometry was used to determine the monosaccharide composition for the leaf, stem and root tissues for two genetic lines of eggplant: (**a**) 305E40; (**b**) 67/3. The y-axis for each graph is discontinuous and shows values from 0 to 90 and 100–500. The error bars represent the standard error between four biological replicates. Ara = arabinose; Rha = rhamnose; Fuc = fucose; Man = mannose; Gal = galactose; GalA = galacturonic acid; Xyl = xylose; GlcA = glucuronic acid; Glc = glucose; GlcN = glucosamine.

GC-MS analysis of the main PCW-associated monosaccharides (Figure 1) revealed that GalA comprised the most abundant monosaccharide in leaf material from both the eggplant lines tested (“305E40” and “67/3”). GalA was also the most abundant monosaccharide detected overall. Despite the especially high standard error values observed for GalA in leaves, sharp decreases in concentration from leaves to stems, and from stems to roots were seen in both varieties. The GalA concentrations (in µg monosaccharide per mg AIR, alcohol-insoluble residue) for leaves, stems and roots in “305E40” were 274.77 ± 193.64, 75.92 ± 24.94 and 21.80 ± 4.35, respectively. Similarly, the GalA concentrations (in µg monosaccharide per mg AIR) for leaves, stems and roots in “67/3” were 328.54 ± 123.82, 58.24 ± 21.68 and 30.39 ± 9.17, respectively. No Fuc was detected in any of the tested samples. Besides Fuc, GlcN was the lowest overall for all samples (<1.06 µg/mg AIR). For both varieties, Ara, Man and Xyl were present in the lowest concentrations in leaf samples when compared to those of stems and roots. The same observation was made for Rha in 305E40 but was absent in “67/3”. Other differences were found among varieties; for example, in 67/3, Ara was more abundant in roots vs. other tissues, while in 305E40 it was more abundant in the stem. It should be noted the genotype comparisons are incomplete because “Black Beauty” was not analyzed using the same vegetative-tissue sampling scheme.

### 2.2. Glycan Array Profiling of Vegetative Organs of Three Eggplant Genotypes

#### 2.2.1. A Direct Comparison of ELISA and CoMPP Analysis and Data-Processing Strategies

**Figure 2:**
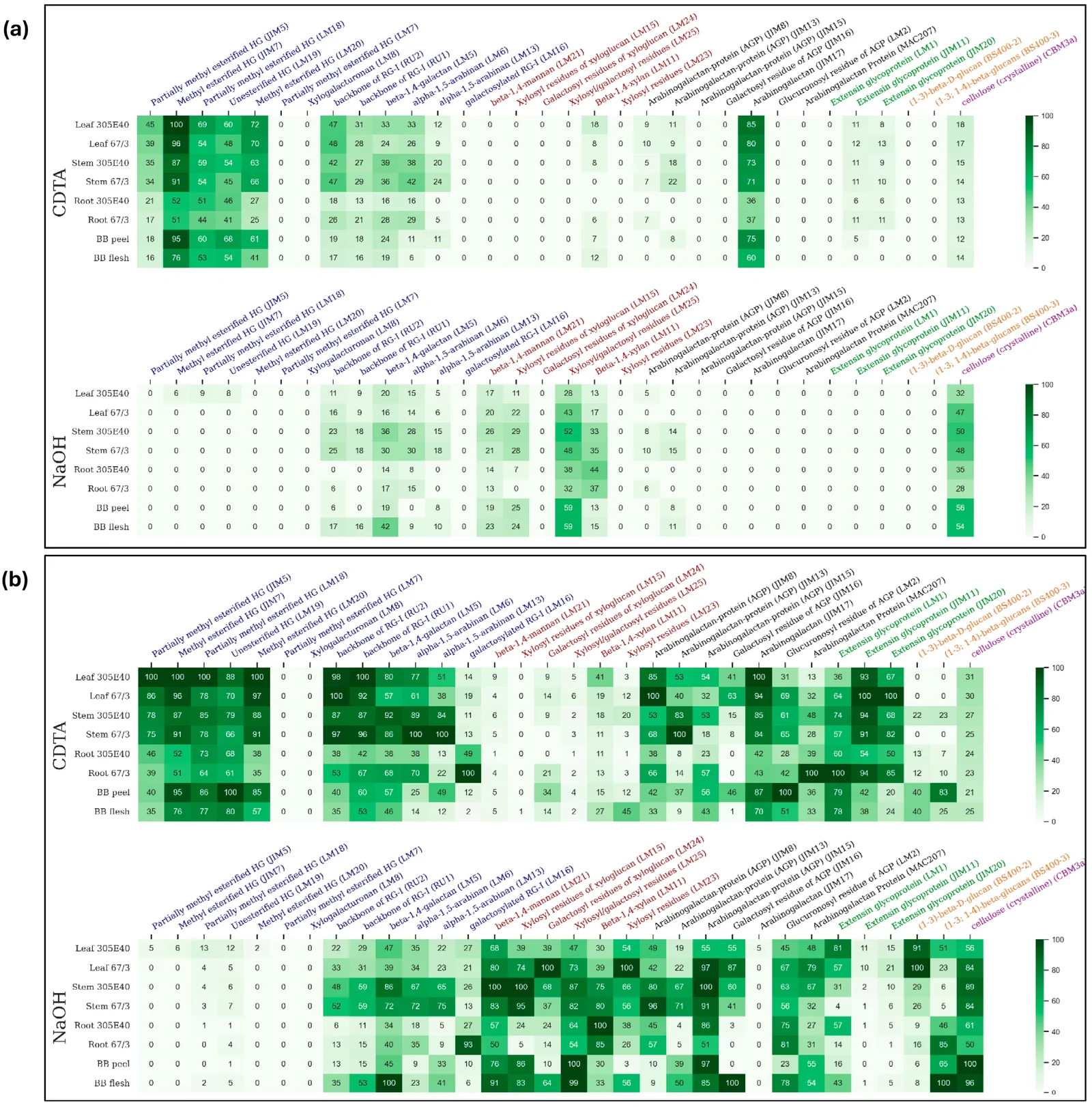
Heatmaps showing two different data-processing strategies for CoMPP data. The relative abundance of selected plant cell wall polysaccharides was compared between three eggplant varieties (Black Beauty, 305E40 and 67/3) and five tissue types (fruit peel (exocarp), fruit flesh (pulp, mesocarp), leaves, stems, and roots) for two chemical fractions: CDTA and NaOH. (**a**) Represents the standard approach for CoMPP data: the maximum value across all columns was assigned a value of 100 and the entire heatmap (for both extractions) was normalized relative to that maximum value. A lower cut-off value of 5 was imposed. (**b**) Shows an alternative strategy for normalizing data where each antibody was normalized individually. No lower cut-off value was imposed. The colored column headings indicate the main polysaccharide/protein classes: blue = pectic probes; red = hemicellulose; black = arabinogalactan proteins; green = extensins; yellow = beta-glucan; purple = cellulose.

**Figure 3:**
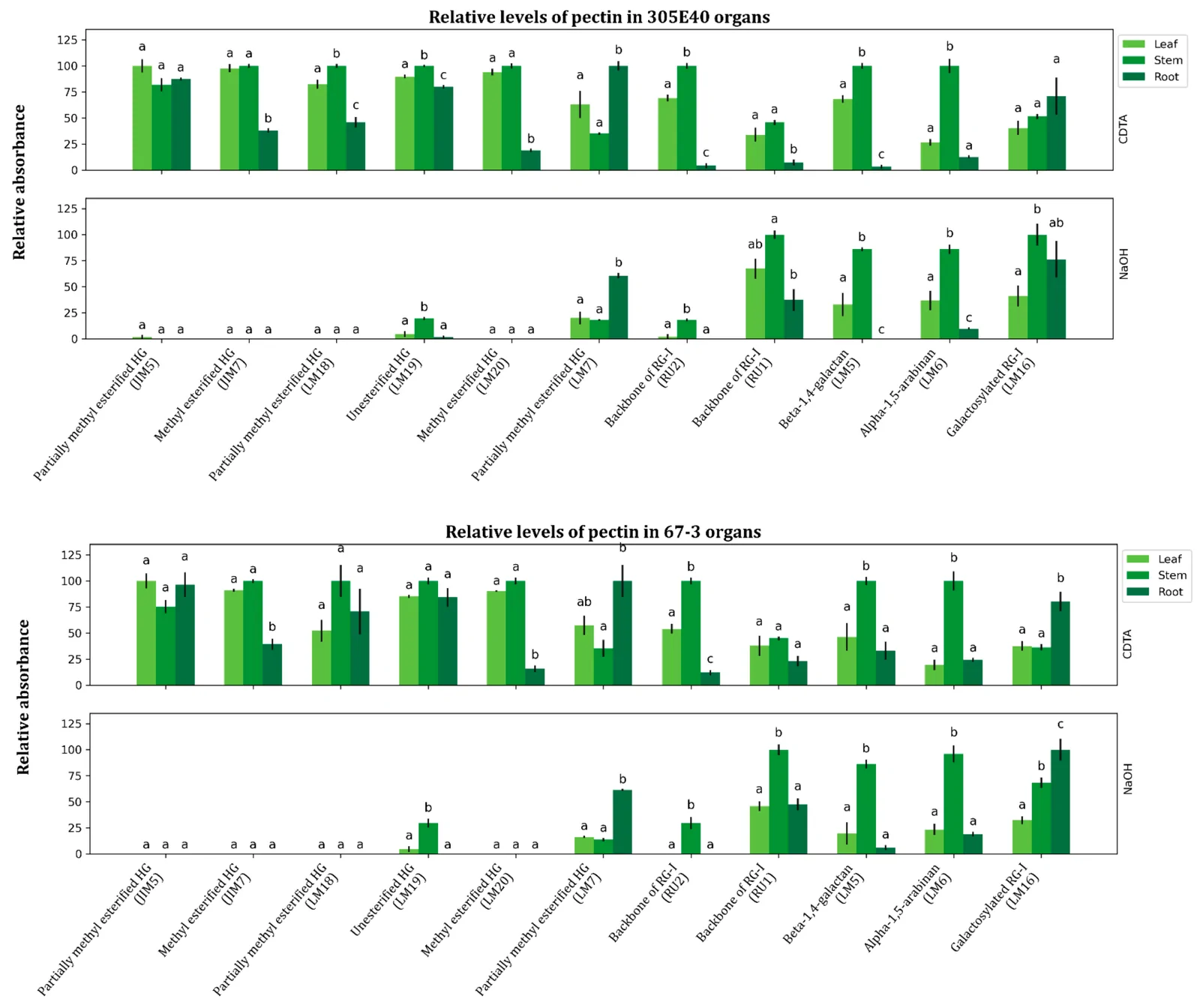
ELISA results for the relative abundance of pectin epitopes in eggplant leaf, stem, and root tissues. Alcohol-insoluble residue obtained from the leaf, stem and root tissues of the eggplant cultivars 305E40 and 67/3 was tested for 11 pectin-associated epitopes using ELISA. Leaf, stem, and root extracts are indicated in light, medium and dark green bars, respectively, and represent relative absorbance values at 450 nm as obtained by ELISA. Statistical significance (*p* < 0.05) (four biological replicates) is shown using letters a to c, and the tissues for each polymer were compared separately. Additionally, the data for each polymer were normalized relative to the maximum value obtained, i.e., the average for the organ that yielded the highest signal for a particular probe was set to 100 and the values for the remaining organs were normalized in proportion. The top and bottom sets of bars for each probe represent sequential extracts of CDTA, followed by NaOH.

A comparison of CoMPP versus ELISA was conducted, as CoMPP provides an overview while ELISA can provide more quantitative data. The use of the terms levels, abundance and relative abundance reflects the higher relative abundance of the corresponding antibody-detectable epitope unless otherwise indicated. CoMPP analysis for all probes was conducted using an average of four sample dilution levels, and a comparison could be made between the obtained signal intensities for 32 glycan-directed mAbs and CBMs (Figure 2). It is important to note that CoMPP analysis was performed in addition to ELISA (shown in Figure 3), mainly to evaluate the different data-normalization strategies, as well as AIR extraction procedures between the two methods. It is important to note the difference between normalization for data visualization, which is mostly used for CoMPP analysis, and ELISA, which commonly employs normalization for quantitative comparisons. However, we only performed semi-quantitative analyses in this study.

The CoMPP signals (Figure 2) for pectic polysaccharides in the CDTA fraction were the highest overall, and leaf tissues of line “305E40” obtained the highest signal across all samples and probes for methyl-esterified HG (JIM7). High values (signals reaching at least 60% of that obtained for JIM7, which was used to normalize the entire dataset) were also obtained for the arabinogalactan probe (JIM17) in the CDTA fraction, especially in leaf, stem and fruit tissues. Most of the signals for the cellulose (CBM3a) and hemicellulose probes (LM11, LM15, LM21, LM25) were observed only in the “strong adherent” 4M NaOH fraction, which is consistent with the fact that very tightly associating CW polymers, especially hemicellulose, are typically extracted under strong alkaline conditions [20].

#### 2.2.2. ELISA Results Compare Root, Stem, Leaf, and Fruit Samples Between Three Eggplant Genotypes

Three eggplant genotypes were compared in two separate experiments: (1) leaf, stem, and root tissues of the “305E40” and “67/3” lines; (2) peel and flesh tissues of store-bought eggplant fruit (“Black Beauty”). It should be noted the ELISA experiments are explicitly divided into vegetative tissues of “305E40”/“67/3” and fruit tissues of “Black Beauty”. Overall, the samples for each of the three genotypes showed the same general trend: pectic and beta-glucan epitopes were mostly present in the CDTA extract, while hemicellulose epitopes were mostly present in the NaOH extract. Antibodies were grouped into three broad classes (pectic polysaccharides, AGPs, and cellulose with hemicellulose) and data for each are presented separately (Figure 3).

##### Pectic Polysaccharides

As shown in Figure 3, HG signals for all samples were mostly present only in the CDTA extraction, except for partially methyl-esterified HG as probed by LM7 (which marks partially methyl-esterified pectin). We also found a signal in NaOH on LM19 (mostly in the stem, unesterified); both findings are likely because the NaOH extracts contained polymers that were more strongly adherent to the primary cell wall (PCW) and were expected to include pectins forming calcium bridges through dimethyl-esterification. The RG-1 and RG-1 sidechain epitopes, on the other hand, were detected in both extractions. For both “305E40” and “67/3”, distinct patterns were observed between the leaf, stem and root samples for several pectin probes (Figure 3). The root samples for both lines showed significantly lower signals when compared to leaf or stem tissues for methyl-esterified HG (JIM7; LM20) and RG-1 backbone (INRA-RU-2). However, partially methyl-esterified HG (LM7) root signals were significantly higher than leaf or stem samples. On the other hand, signals for the RG-1 backbone (INRA-RU2) and the two RG-1 sidechains, β-1,4-galactan (LM5) and α-1,5-arabinan (LM6) were consistently and significantly higher in stem samples than in leaf and root samples for both. One difference between the two lines was evident in their galactosylated RG-1 (LM16) profiles. The root signals of LM16 for “67/3” were significantly higher than leaves and stems for both extractions. In contrast, no significant differences were observed between roots and the other organs, leaves and stems tested.

**Figure 4:**
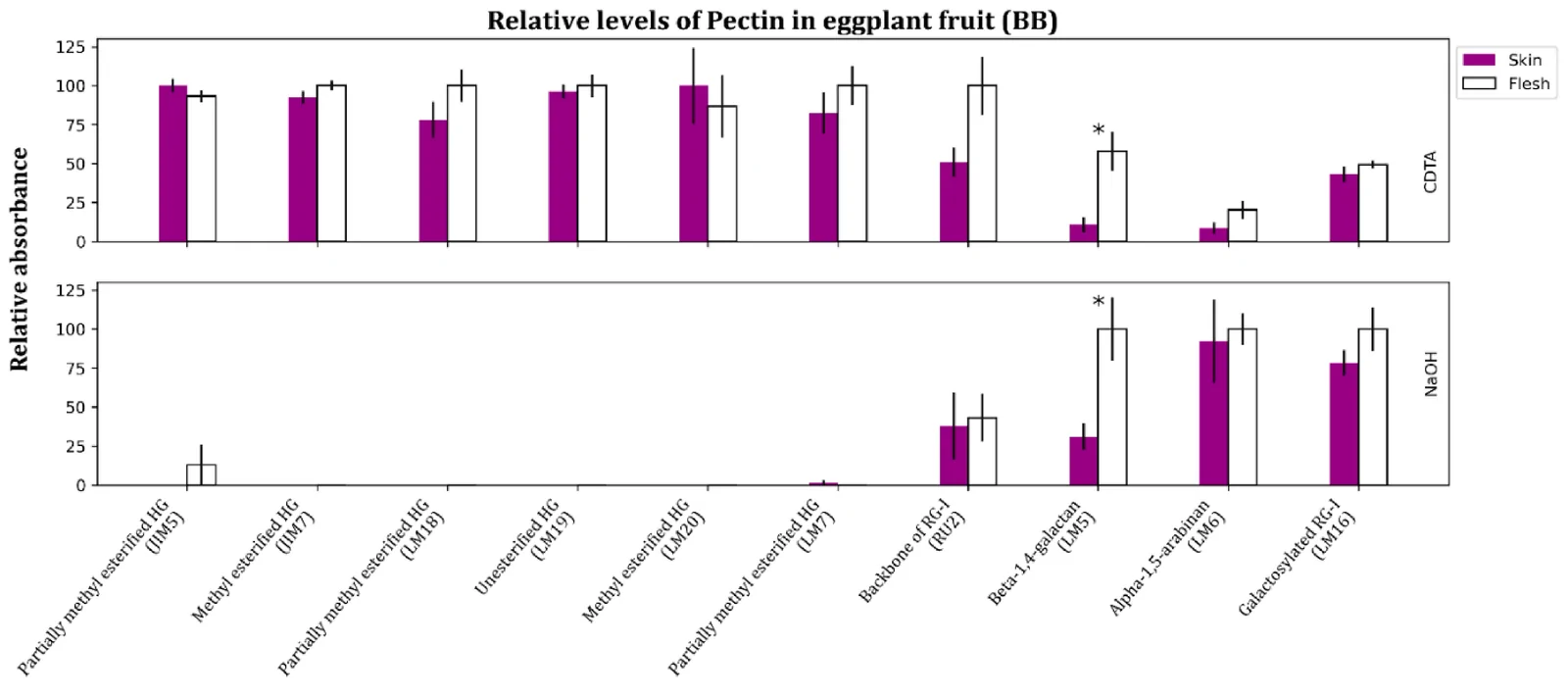
ELISA results for the relative abundance of pectin epitopes in eggplant fruit. Alcohol-insoluble residue obtained from eggplant fruit (cultivar: Black Beauty) was tested for 10 pectin-associated epitopes using ELISA. Skin and pulp samples are indicated as purple and white bars, respectively, and represent relative absorbance values at 450 nm as obtained by ELISA. Statistical differences (*p* < 0.05) (four biological replicates) are indicated with an asterisk, and the tissues for each polymer were compared separately. Additionally, the data for each polymer were normalized relative to the maximum value obtained, i.e., the average for the sample that yielded the highest signal for a particular probe was set to 100 and the values for the remaining samples were normalized in proportion. The top and bottom sets of bars for each probe represent sequential extracts of CDTA, followed by NaOH.

For “Black Beauty” fruit tissues (Figure 4), the only significant difference between peel and flesh samples was observed in β-1,4-galactan (LM5). This should be interpreted in relation to fruit only. LM5 signals were significantly higher in pulp samples for both CDTA and NaOH extractions. Similarly, for all pectic probes aside from partially methyl-esterified HG (JIM5) and methyl-esterified (LM20), flesh samples yielded slightly but not significantly higher average signals when compared to skin samples.

For “305E40” and “67/3,” low unesterified HG (LM19) signals were obtained for stem material in the NaOH extract, while a significant signal for partially methyl-esterified HG (LM7) was detected in root samples in the NaOH extract. Methyl-esterified HG (LM20, JIM7) and RG-1 backbone (INRA-RU2) quantities in the roots of “305E40” and “67/3” were significantly lower compared to leaves and stems. INRA-RU1, also probing for RG-1 backbone, was also less prevalent in root samples of “305E40” compared to leaves and stems, but this was not the case for “67/3”. At the same time, HG that was only partially esterified (LM7) yielded higher signals in roots compared to leaves and stems for both lines. Signals for RG-1 backbone and RG-1 sidechains, β-1,4-galactan (LM5) and α-1,5-arabinan (LM6) were significantly higher in stem samples than leaf and root samples for both lines.

For “Black Beauty” fruit, we detected ten out of the eleven probes used detected in roots, stems and leaves; the only difference was the lack of detection of RU1 signal (RG-1 backbone), which was present in the other three tissues (Figure 3).

##### Arabinogalactan Proteins

**Figure 5:**
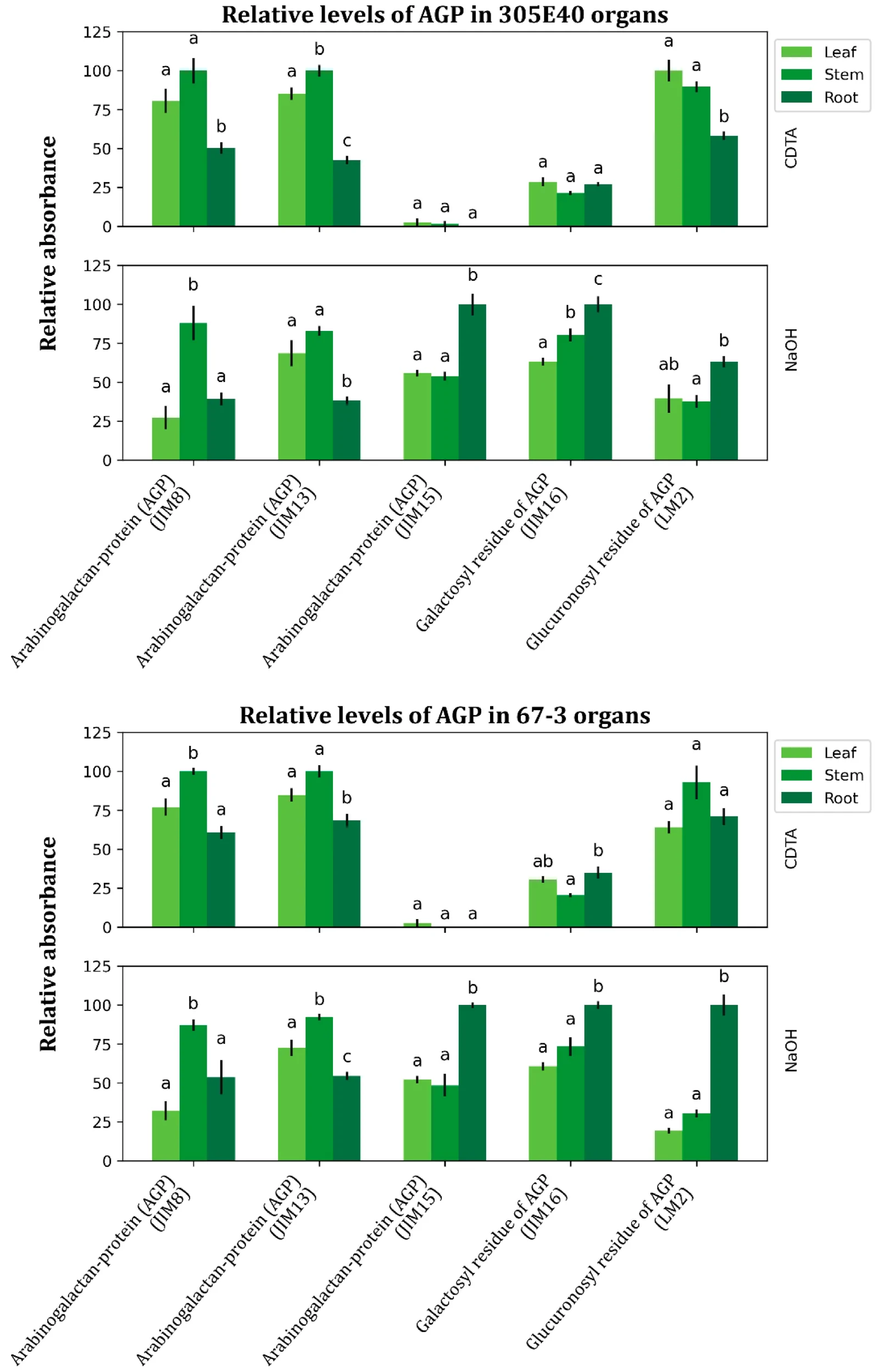
ELISA results for the relative abundance of AGP epitopes in eggplant leaf, stem, and root tissues. Alcohol-insoluble residue obtained from the leaf, stem and root tissues of the eggplant cultivars 305E40 and 67/3 was tested for 5 AGP-associated epitopes using ELISA. Leaf, stem, and root extracts are indicated in light, medium and dark green bars, respectively, and represent relative absorbance values at 450 nm as obtained by ELISA. Statistical significance (*p* < 0.05) (four biological replicates) is shown using the letters a to c, and the tissues for each polymer were compared separately. Additionally, the data for each polymer were normalized relative to the maximum value obtained, i.e., the average for the organ that yielded the highest signal for a particular probe was set to 100 and the values for the remaining organs were normalized in proportion. The top and bottom sets of bars for each probe represent sequential extracts of CDTA, followed by NaOH.

The same general trend in relative AGP signals observed between leaf, stem and root tissues in “305E40,” was also observed in “67/3” (Figure 5). All tissues for these two lines showed signals in both extracts, except for the negligible signals obtained for the AGP probe, JIM15, in the CDTA extract. In the NaOH extract, root samples showed the highest absorbance for JIM15 (AGP), JIM16 (galactosyl residue of AGP) and LM2 (glucuronosyl residue of AGP) compared to leaf and stem tissues, whereas stem samples showed the highest overall quantities of both extractions for the AGP probes, JIM8 and JIM13.

**Figure 6:**
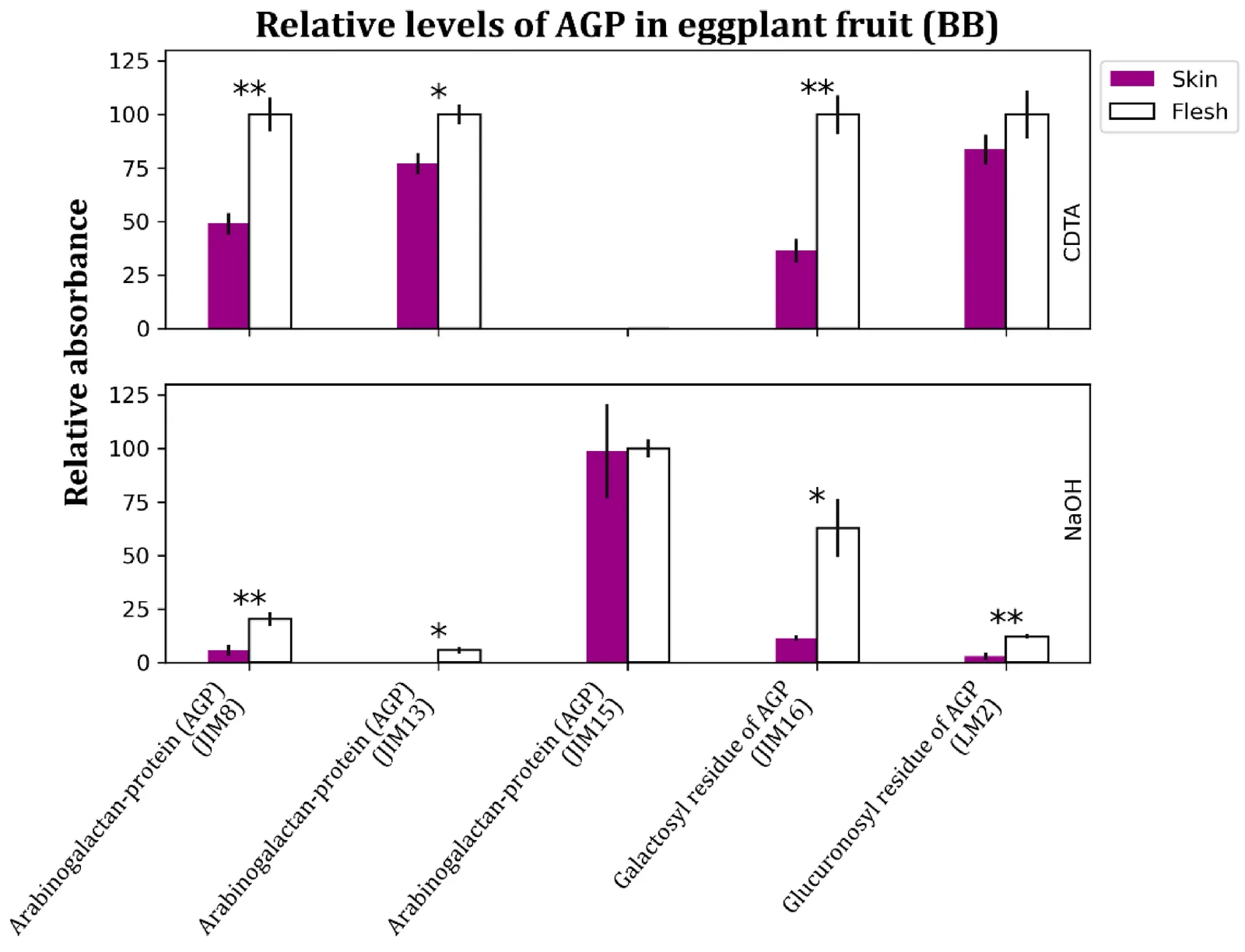
ELISA results for the relative abundance of AGP epitopes in eggplant fruit. Alcohol-insoluble residue obtained from eggplant fruit (cultivar: Black Beauty) was tested for 5 AGP-associated epitopes using ELISA. Skin and pulp samples are indicated as purple and white bars, respectively, and represent relative absorbance values at 450 nm obtained by ELISA. Statistical differences (four biological replicates) for *p* < 0.05 and *p* < 0.01 are indicated with * and **, respectively, and the tissues for each polymer were compared separately. Additionally, the data for each polymer were normalized relative to the maximum value obtained, i.e., the average for the sample that yielded the highest signal for a particular probe was set to 100 and the values for the remaining samples were normalized in proportion. The top and bottom sets of bars for each probe represent sequential extracts of CDTA, followed by NaOH.

For “Black Beauty” fruit (Figure 6), four of the five tested AGP probes showed higher signals in flesh samples than in skin samples. For the AGP probe JIM15, no difference was observed between the two tissue types in NaOH, and no signals for JIM15 were obtained in the CDTA extract, which was consistent with the observations made for “305E40” and “67/3” (leaves, stems, roots) (Figure 6).

When comparing the AGP profile of the three tested genotypes, the solubility characteristics of AGP-epitopes were consistent between “305E40” and “67/3”; however, differences were apparent between these two lines and “Black Beauty” tissue. “Black Beauty” fruits had weak signals in the NaOH extract for LM2 (glucuronosyl residues of AGP), whereas significant signals were obtained for “305E40” and “67/3” tissues. Similarly, while “305E40” and “67/3” showed a significant amount of extracted AGP epitopes in the NaOH fraction, “Black Beauty” fruit yielded low signals in NaOH compared to that of CDTA.

##### Extensins

Extensins were only tested using CoMPP. Under global normalization (Figure 2a), the three extensin probes (LM1, JIM11, JIM20) showed values of roughly 10% for the tissues of “305E40” and “67/3” compared to the global maximum value (JIM7). Overall, “67/3” showed similar or higher values for extensins compared to “305E40”, most prominently in root tissues, where “305E40” had values that were roughly 60% of those of “67/3” for all tissues. “Black Beauty” fruit showed negligible results under this normalization strategy. However, under local normalization (Figure 2b), it was found that “Black Beauty” skin samples yielded similar signals to their corresponding flesh samples for all three extensin probes. The leaf, stem and root samples for “305E40” and “67/3” did not show any trends that were consistent between them. For LM1 in “305E40”, leaves yielded the lowest signals, while roots yielded the lowest signals for JIM11 and JIM20. In the case of “67/3,” LM1 signals were similar in leaves and stems, with a significant increase in roots. For JIM11 and JIM20 in “67/3,” similarly high values were obtained for all tissues, with leaves yielding the maximum values.

Extensins were not tested with ELISA, as we did not have access to the relevant probes; therefore, no comparisons could be made between ELISA and CoMPP for this class of glycoprotein. Overall, CoMPP results showed that “67/3” had stronger signals for each of the three tested extensin probes (LM1, JIM11, JIM20), especially in roots, compared to “305E40” (Figure 2). However, the signals obtained with extensin probes were much weaker in comparison to pectin probes and standard CoMPP visualization (using global normalization, Figure 2a) indicated null signals for all samples tested with mAb LM1, as well as null signals for “Black Beauty” fruit for all extensin probes, with the exception of JIM11 in skin tissues, which had a value of five (the lower cut-off threshold for global normalization). When these data were processed as per our ELISA method (i.e., local normalization per each antibody, Figure 2b), distinct signals were obtained for LM1, JIM11 and JIM20 for all samples tested using CoMPP, emphasizing the potential loss of information when global normalization is applied to glycan-array data.

“Black Beauty” fruit peel and flesh samples showed roughly the same levels of extensins (LM1, JIM11 and JIM20), albeit at low levels, when compared to pectin signals (Figure 2).

##### Cellulose and Hemicellulose

**Figure 7:**
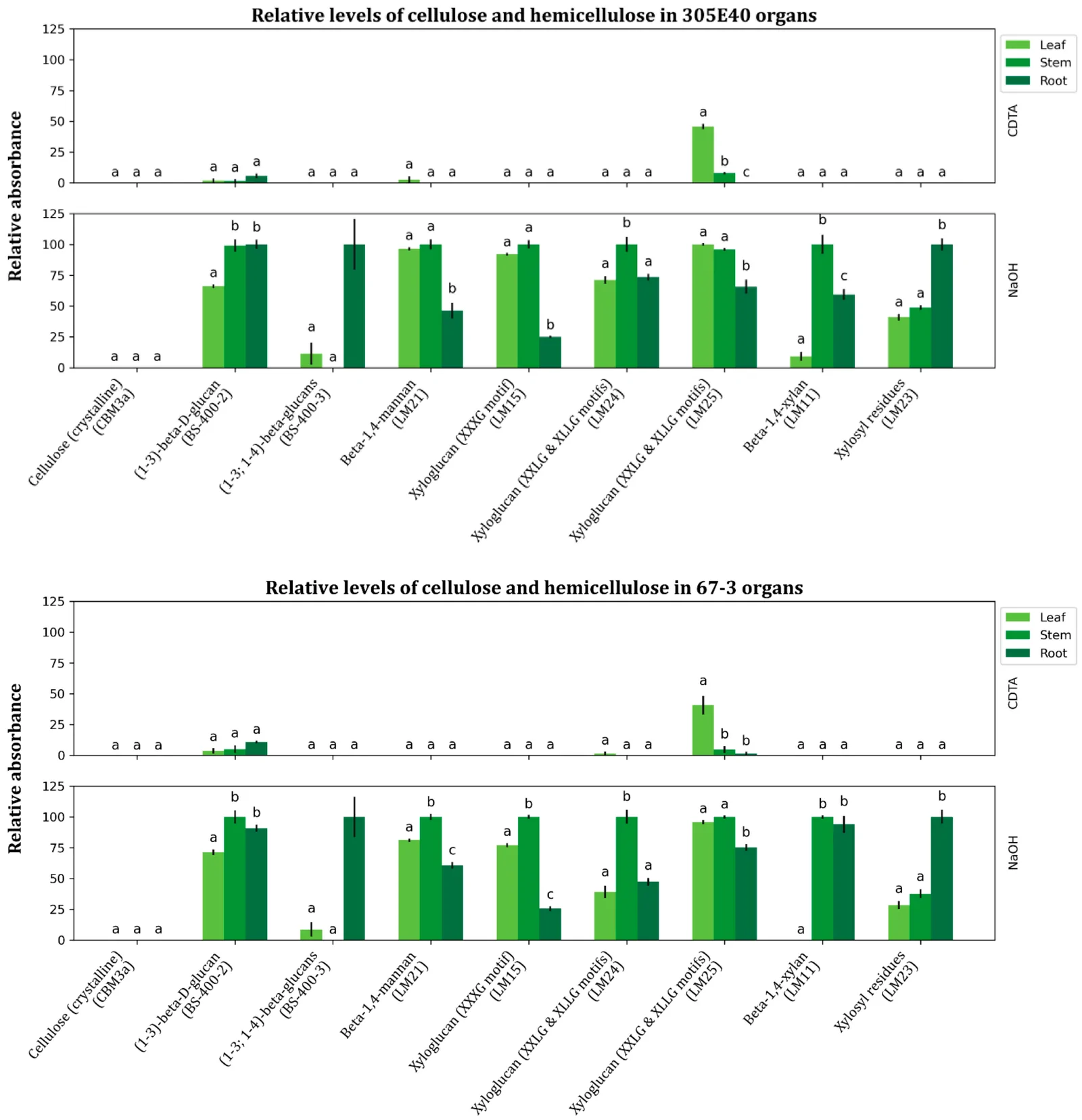
ELISA results for the relative abundance of cellulose and hemicellulose epitopes in eggplant leaf, stem, and root tissues. Alcohol-insoluble residue obtained from the leaf, stem and root tissues of the eggplant cultivars 305E40 and 67/3 were tested for 8 hemicellulose-associated epitopes using ELISA. Leaf, stem, and root extracts are indicated by light, medium and dark green bars, respectively, and represent relative absorbance values at 450 nm as obtained by ELISA. Statistical significance (*p* < 0.05) (four biological replicates) is shown using the letters a to c, and the tissues for each polymer were compared separately. Additionally, the data for each polymer were normalized relative to the maximum value obtained, i.e., the average for the organ that yielded the highest signal for a particular probe was set to 100 and the values for the remaining organs were normalized in proportion. The top and bottom sets of bars for each probe represent sequential extracts of CDTA, followed by NaOH.

For “305E40” and “67/3” (Figure 7), most of the cellulose and hemicellulose signals were obtained from the NaOH fractions. The notable exception was the leaf samples in both lines, where these signals were present at significant levels in the CDTA fraction for xyloglucan (XXLG/XLLG motifs; LM25). Root samples for both lines yielded higher absorbance between the three tissue types for (1-3, 1-4)-β-glucans (BS-400-3) and xylosyl residues (LM23). On the other hand, root signals were the lowest of the three tissue types for β-1,4-mannan (LM21) and the XXXG motif of xyloglucan (LM15). No detectable signals could be obtained for stem samples using BS-400-3 to probe for (1-3, 1-4)-β-glucans. Similarly, negligible levels of signal were obtained for BS-400-3 and β-1,4-xylan (LM11) leaf samples. No signal was obtained for the crystalline cellulose/xyloglucan probe, CBM3a (Figure 7). This observation contrasts with the data obtained from CoMPP, which showed similar levels in the leaves and stems of both “305E40” and “67/3”, with slightly lower levels in their respective root samples (Figure 2).

**Figure 8:**
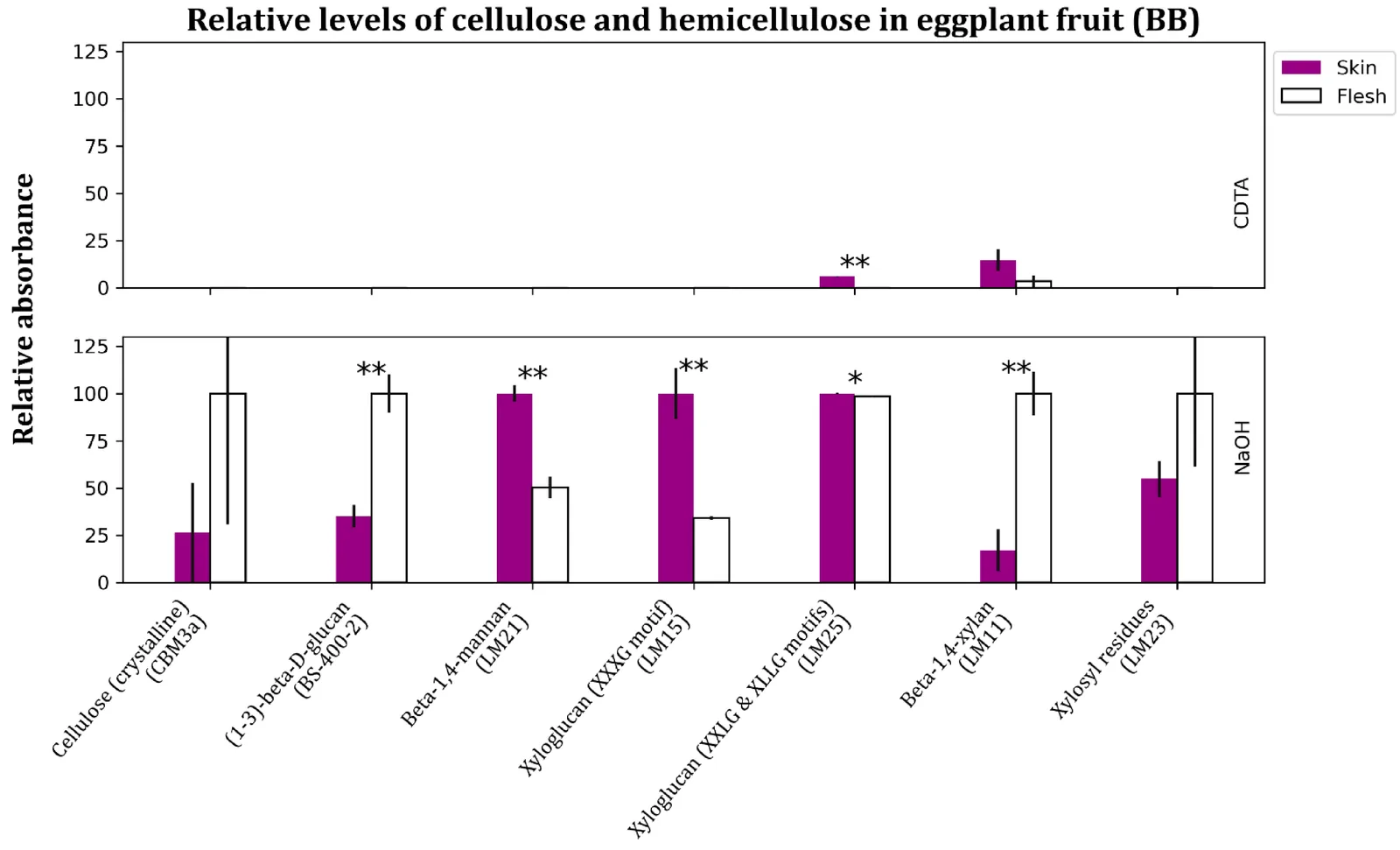
ELISA results for the relative abundance of cellulose and hemicellulose epitopes in eggplant fruit. Alcohol-insoluble residue obtained from eggplant fruit (cultivar: Black Beauty) was tested for 6 hemicellulose epitopes using ELISA. Skin and pulp samples are indicated as purple and white bars, respectively, and represent relative absorbance values at 450 nm obtained by ELISA. Statistical differences (four biological replicates) for *p* < 0.05 and *p* < 0.01 are indicated with * and **, respectively, and the tissues for each polymer were compared separately. Additionally, the data for each polymer were normalized relative to the maximum value obtained, i.e., the average for the sample that yielded the highest signal for a particular probe was set to 100 and the values for the remaining samples were normalized in proportion. The top and bottom sets of bars for each probe represent sequential extracts of CDTA, followed by NaOH.

For “Black Beauty” fruit (Figure 8), all tested epitopes were detected in the NaOH extract. However, only xyloglucan (XXLG/XLLG motifs; LM25) and β-1,4-xylan (LM11) were detected in the CDTA extract, and in small amounts. Significant differences were observed between skin and flesh samples for all hemicellulose probes except for xylosyl residues (LM23). Skin samples yielded stronger signals for β-1,4-mannan (LM21), xyloglucans (XXXG motifs; LM15, XXLG/XLLG motifs; LM25). On the other hand, flesh yielded a higher number of signals for (1,3)-β-D-glucan (BS-400-2) and β-1,4-xylan (LM11). For LM11, a roughly equal number of signals were obtained for the CDTA and NaOH extractions of skin samples. However, negligible signal was obtained in the CDTA extract from flesh tissue, whilst a significant signal was detected in the NaOH extract. Lastly, signals for crystalline cellulose/xyloglucan (CBM3a) were detected in “Black Beauty” fruit (Figure 8), unlike material from “305E40” or “67/3”. In contrast, the CoMPP analysis of pooled samples indicates that similar levels of epitopes for CBM3a were detected in skin and flesh samples, with relative values of 56 and 54, respectively (Figure 2).

## 3. Discussion

To our knowledge, this is the first study to compare the polysaccharide composition between eggplant exocarp and mesocarp tissues [11,21].

### 3.1. Monosaccharide Identification and Quantification by GC-MS

For the “305E40” and “67/3” lines, the highest concentrations for GalA and Glc were observed across all monosaccharides in the leaf tissue. Nguema-Ona et al. [22] chemically fractionated AIR prepared from fully expanded tobacco leaves (de-starched) and also observed that GalA was the most abundant monosaccharide overall. This is consistent with abundant literature data showing pectin as the major component of the primary cell walls in dicotyledonous plants, constituting up to 35% of the wall’s composition [23]. In *Arabidopsis thaliana* leaves, pectin content can be approximately 50% by weight, though this varies depending on environmental factors, tissue type, and species. Pectin’s abundance and composition are crucial for various plant physiological processes, including cell adhesion, growth, and response to environmental stimuli. While our study made use of only chelator (CDTA [50 mM]) and alkaline (NaOH [4 M]) extractions, Nguema-Ona and colleagues made use of sequential extractions using a hot buffer (100 °C sodium acetate), a chelator (CDTA [1% *w*/*v*]), Na_2_CO_3_ extract and an alkaline extraction (KOH [4 M]). GC-MS analysis of AIR powder as well as chemical fractions revealed that GalA had the highest abundance by a significant margin in all fractions except the alkaline extract. The abundance of GalA observed in our study in the AIR of “305E40” and “67/3” likely reflects a high abundance of pectin polymers, since GalA comprises the backbone of homogalacturonan (HG) as well as a major component of the RG-1RG-1 backbone [24]. Our CoMPP results corroborated this finding, as HG showed the highest number of signals overall for eggplant leaf, stem and root AIR, followed by RG-1 (Figure 2). This is a fair comparison in this case, since all samples were tested at the sample concentrations using CoMPP after which the entire dataset was normalized to the highest values obtained. However, for our ELISA results, the data for each probe should be evaluated individually, since an optimal concentration window was used for each probe. Our CoMPP results showing the highest pectin values in leaf tissues relative to stems and roots (using JIM5, JIM7, LM18, LM19 and LM20) support our monosaccharide data in indicating that pectin may be the most abundant polysaccharide (besides cellulose) in our analyzed samples, especially in leaves. In contrast to the observations for GalA, leaf samples for both varieties showed the lowest concentrations compared to stems and roots for Xyl. Both CoMPP (Figure 2) and ELISA data (Figure 7) show that xylan (mAb LM11), consisting of β-1,4-linked Xyl [25], was the lowest in leaf tissues compared to stems and roots. Low Xyl signals in leaves may explain the low concentrations of Xyl observed in leaves using GC-MS.

### 3.2. Glycan Array Profiling of Vegetative Organs of Three Eggplant Genotypes

#### 3.2.1. A Direct Comparison of ELISA and CoMPP Analysis and Data-Processing Strategies

When comparing the data-processing strategies for CoMPP (global normalization, Figure 2a) and our ELISA method (local normalization, Figure 2b), several differences became apparent, the first of which was the suggestion of a lack of detectable signals created when normalizing glycan microarray data globally across several antibodies. Figure 2a shows null values for 12 probes (LM7, LM8, LM16, LM24, LM23, JIM15, JIM16, LM2, MAC207, BS-400-2, BS-400-3), 10 of which showed signal (all except for LM7 and LM8) when each column was normalized individually (as per our ELISA method) without imposing a lower cut-off threshold of five (Figure 2b). The probes for galactosylated RG-1RG-1 (LM16), xylosyl residues (LM23), glucuronosyl residue of AGP (LM2) and (1-3; 1-4)-beta-glucan (BS-400-3), while displaying null values under global normalization (Figure 2a), showed clear patterns between sample types when normalized individually (Figure 2b). In these cases, significant data was swamped due to some probes yielding higher overall signals when normalizing globally. In other words, signals (which correlate to epitope abundance) that were less than 1% compared to the global maximum (JIM7 in 305E40 cultivar epitope signal) were drowned out, since this approach assigns the maximum value in the dataset a value of 100 and the rest of the dataset is normalized accordingly. This threshold is even higher in cases where a lower cut-off value is established—commonly 5% of the global maximum value [18].

CoMPP was used in addition to our ELISA protocol to serve two purposes: (1) to compare our own developed method to a well-established methodology [18,26], and (2) to evaluate the data-processing and visualization strategies used in our ELISA method against those of CoMPP. Similar trends were observed for most probes between ELISA and CoMPP when looking at root, stem and leaf samples for “305E40” and “67/3”, as well as the peel and flesh of “Black Beauty”. While some differences could be attributed to different data-processing approaches, other differences could be attributed to inherent differences in sample handling and probing. For example, we were not able to obtain a usable signal for the crystalline cellulose/xyloglucan probe (CBM3a) in any of our experiments, whereas during the CoMPP analysis, clear tissue-specific distribution patterns were seen (Figure 2) for the same samples in both the CDTA and NaOH fractions. This result could reflect the impact that different extraction procedures may have on the detectible epitopes present in each chemical fraction, especially the impact on cellulose extraction [26,27]. Cadoxen (cadmium oxide with diaminoethane) can be used as a third extraction after the sequential CDTA and alkaline extraction steps to gain access to cellulose epitopes [28]. The fact that we did not include a cadoxen extraction in our ELISA-based method could explain why it yielded no apparent signal for CBM3a [22,26].

In terms of data processing, the use of global normalization as opposed to local normalization when processing microarray data can impact the interpretation of the results (Figure 2). Global normalization is the standard CoMPP visualization strategy, where the maximum signal for all antibodies is set to 100 and all other signals are normalized in relation to this (Figure 2a). This strategy is best reserved for quantitative data, where a standard calibration curve can be used to quantify the signals obtained for each probe due to varying avidities observed between probes [29,30]. Global normalization may cause the signals of strongly binding probes (or a high abundance of epitopes for that probe in a sample) to drown out lower signals, since a window of two orders of magnitude in addition to CoMPP’s lower cut-off value of five may not represent lower signals in the final heatmap (Figure 2a). By normalizing the CDTA and NaOH data for each probe individually (Figure 2b), epitopes are represented on the heatmap that would otherwise have been interpreted as absent (Figure 2a). The consequence of globalized normalization was that 10 probes (BS-400-2, BS-400-3, LM1, LM2, LM16, LM23, LM24, JIM15, JIM16, MAC207) were assigned null values instead of usable data (Figure 2a). Additionally, a low number of signals were recorded for AGP epitopes (JIM8, JIM13), which may have led to this polysaccharide class being under-represented in evaluations of the cell wall profile of a plant, since the lower relative numerical difference between signals may have hindered effective interpretation. Therefore, the local normalization method (Figure 2b) may be a useful alternative to the method conventionally used for CoMPP data, especially in the case of using heatmaps for data visualization.

#### 3.2.2. ELISA Results Compare Root, Stem, Leaf, and Fruit Samples Between Three Eggplant Genotypes

##### Pectic Polysaccharides

Our results supported the presence of 11 pectin-associated epitopes in eggplant leaf, stem, root and fruit material (Figure 3). Most HG epitopes were detected in the CDTA fraction. In contrast to HG, RG-1RG-1-related probes were detected in both extractions. Significantly higher numbers of stem sample signals were consistently detected for the RG-1RG-1 backbone (INRA-RU2) and the two RG-1RG-1 sidechains, β-1,4-galactan (LM5) and α-1,5-arabinan (LM6). It is possible that, as tissues mature, modifications in galactan and arabinan content might influence the structural integrity of cell walls. These polymers could potentially contribute to the flexibility and extensibility of cell walls, which may be important during growth and differentiation. The number of root signals of LM16 in “67/3” was significantly higher than in leaves and stems for both extractions, whereas no significant differences were observed for “305E40”. Given that galactosylated RG-1RG-1 participates in interactions with other cell wall components, enhancing tissue cohesion and mechanical integrity, there may be varietal differences in how these properties manifest across tissues “305E40”.

The results for “Black Beauty” fruit tissues (Figure 4) were consistent with the known role of galactans in contributing to the structural integrity and stability of the cell wall, as well as in water retention, which is crucial for maintaining fruit weight and influencing the firmness and texture of fleshy fruits.

The results from this study revealed distinct patterns in the pectic polysaccharide profiles of the three tested eggplant genotypes, “305E40”, “67/3” and “Black Beauty”. The degree of methyl-esterification of pectin as well as the pH affect the solubility of pectin [31,32]. Non-ionic pectic polymers bound to the cell wall matrix non-covalently are typically water-soluble; pectins that are bound via ionic bonds (e.g., eggbox structures in which HG chains are bound via Ca^2+^ ions) are soluble in the presence of a chelator (such as CDTA), and pectins that bind to the cell wall matrix via covalent ester bonds are typically soluble in alkaline environments [31,32]. Overall, our results indicate that the distribution of pectic polysaccharides is mostly consistent between skin and flesh samples for “Black Beauty” fruit.

Our data on the different accumulation patterns of pectin types (LM19, LM20) and galactosylated RG1 (LM16) in EDTA/NaOH extracts align with the changes in accumulation patterns reported by Kutyrieva et al. recently [33]. There, the authors observed alterations in the deposition of these epitopes during tomato fruit ripening in specific lines with modifications to the ripening process. These findings suggest that alterations in pectin homeostasis are critical for regulating fruit stiffness during ripening. Our test with different chemical treatments to extract various adherent-associated layers highlights how these treatments reveal differences in pectin composition and its role in regulating fruit texture. We hypothesize that specific alterations in the pectin network—particularly in the balance between partially methyl-esterified homogalacturonan (LM19 and LM20), as well as galactosylation of RG1 (LM16)—may directly influence fruit firmness and texture during ripening. Future studies could test whether manipulation of these pectin components by specific genetic or biochemical approaches can modulate fruit firmness and postharvest quality. This could provide valuable information on possible strategies to improve fruit texture in commercial cultivation.

This is especially surprising if one considers how the consistencies differ between “Black Beauty” fruit skin (which is smooth and firm) and flesh (which is softer and spongey). The pectin differences may contribute to texture differences, but this is only hypothetical in the absence of experimental datasets on firmness. More genotypes need to be analyzed to determine whether this finding holds for eggplant materials other than “Black Beauty”. Studies on eggplant cell walls are limited and typically focus on whole fruits instead of skin and flesh analyzed separately [8,9,10,13,21].

Overall, our results support the presence of 11 pectic epitopes in the cell wall extracts of all three genotypes and highlight the relative abundance of said epitopes in different organs. The distinct patterns in polysaccharide abundance observed in eggplant organs suggest that the biosynthesis and deposition of pectic polysaccharides likely vary considerably between organs [31].

##### Arabinogalactan Proteins

The tissue distribution of AGP epitopes was mostly consistent for the leaves, stems and roots between the two lines, “305E40” and “67/3”. AGPs are localized in several locations in a cell, including the cell wall, plasma membrane and soluble extracellular exudates [34]. These proteins have been described as ideal mediators between the cytoplasm, plasma membrane and cell wall matrix due to their amphiphilic nature [35]. The high AGP epitope signals in “Black Beauty” fruit flesh relative to skin samples may reflect the structural functions of AGPs and may hypothetically suggest closer associations between AGPs and other cell wall polymers in the tough eggplant skin tissues, which ultimately result in lower solubility of AGPs in the CDTA and NaOH extracts of skin samples [36,37,38]. An additional hypothesis is that the stronger association of AGPs in fruit flesh vs. skin CW is linked to the higher content of galactan sidechains detected in fruit flesh. Galactan-rich sidechains may facilitate tighter interactions with cellulose and hemicellulose networks (see the upcoming discussion on differences between flesh and skin tissues) or with other matrix polysaccharides, such as pectins, through hydrogen bonding or other non-covalent interactions. Future studies should include several eggplant genotypes to determine whether this finding is consistent in eggplant germplasm; moreover, it would be interesting to estimate the environmental effects on their levels and tissue-level distribution.

##### Extensins

Extensins are cell wall-associated hydroxyproline-rich glycoproteins. The biological functions these proteins are involved in are numerous, including growth and maintenance of the cell wall, cell wall assembly, defense against microbial pathogens, and aiding in maintaining cell-wall structure [39]. To aid in structural support, the cross-linking of PCWP may help to disperse the load stress experienced by the cell wall [40]. Extensins have been shown to cross-link with pectin [41], and evidence for cross-linkages of extensins with AGPs and lignin has also been proposed [38,42,43].

Due to the magnitude of roles played by extensins in the cell wall, it is not possible, based on the present results, to give a definitive explanation as to why one eggplant line would have significantly higher levels of extensins in leaves, stems, and roots [39]. Although, according to Castilleux et al. [44], Lamport et al. [39] and Kieliszewski and Lamport [45], a higher resistance of “67/3” to pathogenic attacks should be expected due to its high relative abundance of these glycoproteins compared to “305E40”, actually, this latter line displayed a significantly improved resistance to the vascular fungal wilts caused by *Fusarium oxysporum* f. sp. *melongenae* and *Verticillium dahlia*. In fact, segregating populations from the cross between “305E40” and “67/3”are being usefully exploited to identify QTLs and candidate genes underpinning the improved resistance displayed by “305E40” lines [46,47]. Regarding protection against pathogenic attacks, Castilleux et al. [44] highlighted the defensive roles extensins take on during plant–pathogen interactions—not only in the cell walls, but in secretions in the extracellular mucilage of plant roots. Extensins have even been shown to be secreted in higher concentrations in extracellular mucilage during an immune response to root pathogens [48].

The observed LM1 signal in fruits aligns with recent findings reporting high levels of extensin epitopes in ripening tomato fruits [33]. These findings, based on immunocytochemical assays, revealed stage-specific differences in extensin accumulation during the ripening process, with visible variations between distinct ripening stages.

##### Cellulose and Hemicellulose

Cellulose is arranged into microfibrils. In contrast, hemicelluloses are composed of shorter chains which may feature branching. Hemicelluloses can be defined broadly as polysaccharides that feature β-1,4-linked backbones and that are classified as neither pectin nor cellulose [49]. These polysaccharides can be categorized into the general classes: xyloglucan, xylan, mannan and mixed-linkage β-glucan [50]. Overall, no signals were obtained for crystalline cellulose (CBM3a) in the CDTA or NaOH fractions for any of the tested leaf, stem and root samples. A third extraction on the AIR with cadoxen may have been able to extract and solubilize cellulose [18,51]. Signals were obtained for “Black Beauty” skin and flesh samples; however, the signals were below the window of quantification and very high variability was observed between biological replicates. Thus, no conclusions can be drawn from CBM3a data in eggplant fruit. In terms of hemicellulose, most of the signals obtained were in the NaOH fraction for all eggplant material tested (Figure 8). Since the high pH of the NaOH fraction—or “hemicellulose-rich fraction”—is known to extract hemicellulose, this observation was not unexpected [18]. However, LM25 (probing for XXLG and XLLG motifs of xyloglucan) yielded significant signals in the leaves of all three tested genotypes. Among the xyloglucan-directed probes, LM24 and LM25 [52] displayed an affinity for a larger array of galactosylated xyloglucan oligomers than the previously profiled probe, LM15 [53]. All three of these probes yielded significant signals in the NaOH fraction of all tested samples. However, only LM25 signals in eggplant leaves featured prominently in their respective CDTA fractions. In addition to xyloglucan, LM25 has been shown to exhibit weak binding to unsubstituted β-glucan; however, neither of the two tested β-glucan probes (BS-400-2 and BS-400-3) yielded prominent signals in the CDTA fractions. LM15 and LM25 signals have been detected in whole homogenized eggplant fruits in the alkaline fraction of AIR [11]. Interestingly, these signals were only observed in eggplant fruits and not in tomato, strawberry or apple. Our study supported the presence of these epitopes in “Black Beauty” fruits. Since our study discriminated between fruit skin and flesh tissues, we proposed that the vast majority of LM15’s target epitopes (XXXG motif of xyloglucan) were present in the fruit skin. Signals for LM25 were also significantly higher in fruit skin. In a study comparing different solanaceous fruits, significant structural homology was observed between the xyloglucan contents of eggplant, tomato, red pepper and sweet pepper fruits [12].

The xylan mAb LM11 has been shown to bind to arabinoxylan, low-substitution xylans, and glucuronoarabinoxylan [54]. LM11 has documented binding specificity towards particular xylan/arabinoxylan-related structural epitopes [54]. Xylans are especially abundant in plant secondary cell walls, and heteroxylans are considered to be the main polysaccharides apart from cellulose in the secondary PCW of lignified angiosperm tissues [55]. Lignification allows for effective water transport and rigidity in vascular plants [55]. Therefore, the high relative abundance of xylan epitopes observed in our experiments for stem and root cell walls in comparison to leaves (Figure 7) is in line with what is expected of rigid load-bearing organs containing mature secondary cell wall-rich vasculature tissues [56]. Additionally, it has been shown in dicotyledonous plants that mAb LM11 epitopes are restricted to secondary cell walls [54]. The negligible signals for LM11 in “305E40” and “67/3” leaves (fully mature), compared to high numbers of signals in stems and roots, support the finding that xylan levels are low in mature eggplant leaves. LM11 signals were obtained for “Black Beauty” fruit, although mostly in flesh tissues. Eggplant fruits have been found to contain a relatively large amount of xylan epitopes (probed for by LM11 and LM28), while tomato, strawberry, and apple fruits contained no detectable epitopes [11]. Nevertheless, the high LM11 signal observed in our study aligns with findings by Kutyrieva-Nowak et al. [33], who demonstrated through xylan immunocytochemical labeling that the ripening process in tomato fruits is associated with significant changes in xylan deposition. Their assays revealed that these xylan modifications are linked to changes in the expression of the *SlP4H3* gene (a prolyl-4-hydroxylase) and alterations in the fruit maturation process.

This study made use of whole homogenized fruits. However, our study drew a distinction between eggplant skin and flesh, and our results show that the vast majority of LM11 epitopes were localized in flesh tissues.

## 4. Materials and Methods

### 4.1. Plant Material

Eggplant fruit of the cv “Black Beauty” was obtained from a local supermarket (Woolworths, Stellenbosch, South Africa, dated 15 October 2022) and stored at 4 °C for 3 d before processing. The sampled vegetative tissues of the two parental lines “305E40” and “67/3” of a RIL population were obtained from the Council for Agricultural Research and Economics (CREA), Research Centre for Genomics and Bioinformatics, Montanaso, Lombardo (LO), Italy. In analyzing these samples, the authors treated each as separate experiments, and glycan-array data were only compared within each experiment.

### 4.2. Generation of Cell-Wall Material

Fruits were flash-frozen immediately after separating the skin (exocarp) and flesh (mesocarp, placenta and core, de-seeded with a small knife) to avoid the oxidation of phenolic compounds. This represents the characterization of supermarket-derived “Black Beauty” fruit under the stated postharvest conditions. A potato peeler (Victorinox Swiss Made Stainless Steel Swiss Classic Ypso Peeler, Ibach, Schwyz, Switzerland) was used to separate the peel from the flesh. The generation of alcohol-insoluble residue (AIR) from PCWs was performed as described by Moore et al. [57]. The analyses of “Black Beauty” fruit, “Black Beauty” leaves, and material from the 2 breeding lines were treated as separate experiments and glycan-array data were only compared within each experiment. Statistical inference was based on biological replicates and that technical replicates were averaged before inferential testing was conducted experimentally.

### 4.3. Gas Chromatography–Mass Spectrometry for Analyzing Monosaccharides

Gas chromatography–mass spectrometry (GC-MS) was used to quantify the relative levels of monosaccharides that make up the AIR of eggplant leaves, stems, and roots. GC-MS was only applied to AIR from “305E40” and “67/3” and samples were prepared and analyzed according to the protocol followed in Nguema-one et al. [22]. The monosaccharides analyzed included glucose (Glc), fucose (Fuc), galactose (Gal), xylose (Xyl), galacturonic acid (GalA), glucuronic acid (GlcA), arabinose (Ara), mannose (Man), rhamnose (Rha), glucosamine (GlcN) and myo-inositol. All monosaccharide standards (≥99% purity) were purchased from Sigma-Aldrich (Steinheim, Germany).

### 4.4. Chemical Extraction of AIR Material

AIR was weighed out into microcentrifuge tubes. Two small metal ball bearings were added to each tube to aid in homogenizing the AIR before the subsequent chemical extraction. Two rounds of chemical extraction were performed, using CDTA (50 mM in Tris, pH 7.2) and NaOH (4 M containing 0.1% NaBH_4_ [58].

### 4.5. Optimized Enzyme-Linked Immunosorbent Assay

Enzyme-linked immunosorbent assay (ELISA) was performed on sequential 50 mM CDTA and 4M NaOH extracts of plant AIR using the 24 monoclonal antibodies (mAbs) and one carbohydrate-binding module (CBM) listed in Table 1. mAbs were obtained via different suppliers; BS-400-2 and BS-400-3 were obtained from Biosupplies Bundoora, Australia; INRA-RU1 and INRA-RU2 were obtained from INRAE, France; LM and JIM mAbs were obtained from Kerafast (Kerafast, Inc., Boston, MA, USA). Horseradish peroxidase-conjugated secondary antibodies were obtained from Biocom Africa (Pty) Ltd., Centurion, South Africa, and included donkey anti-mouse IgG (heavy and light chain), goat anti-rat IgG (heavy and light chain), goat anti-rat IgG2a, goat anti-rat IgG2c, goat anti-rat IgM (heavy chain), and goat anti-rat IgA (heavy chain).

An ELISA protocol was developed for throughput and cost following the Glycome Profiling method outlined by Pattathil et al. [75] and the derivative method, gel-permeation chromatography–ELISA [27] as references. ELISA analyses were conducted in Costar 96-well tissue culture-treated plates (Costar 3598, Corning, NY, USA), following the protocol outlined by Sathitnaitham et al. [27]. Preliminary testing informed the optimal testing concentration for each antibody by testing a dilution series of a pooled representative sample of all the samples to be tested using ELISA. After the optimal concentrations were determined, samples were diluted to their optimal concentration, and 50 µL aliquots of each sample were transferred in triplicate to the 96-well plates before the plates were incubated while uncovered at 37 °C to dry overnight. The plates dried within 2–3 h but were incubated overnight for convenience. Blocking protein (3% bovine serum albumin [BSA] in phosphate-buffered saline [PBS, 9.55 g/L in dH_2_O]) was added to each well (200 µL) and the plates were stored covered at room temperature at 22 °C for 8 h. After blocking the BSA solutions were discarded, and the primary antibodies were added in 30 µL aliquots, diluted 1:120 in 1% BSA in PBS (9.55 g/L), after being allowed to reach room temperature. The plates were incubated at room temperature in a sealable ice box to limit airflow and potential temperature gradients over the plates. After incubation, the plates were washed three times using diluted PBS (4.78 g/L). Each wash entailed using a wash bottle to overfill each well with PBS buffer and discarding the buffer by forcefully swinging the plate into a waste container and using the flick of one’s wrist to expel as much liquid from the plate as possible. After three washing steps, the relevant secondary antibody was added in 50 µL aliquots diluted 1:20 000 in 1% BSA in PBS. Six washes (PBS, 4.78 g/L) followed to ensure the removal of all unbound antibodies. The chromogenic substrate 3,3′,5,5′-tetramethylbenzidine was prepared 10 min before use and added to each well in aliquots of 75 µL (0.42 mM). The resulting chromogenic reaction was terminated by adding 125 µL of 1 M H_2_SO_4_ to each well after exactly 30 min. Absorbance readings were taken at 450 nm as soon as possible to prevent non-enzymatic oxidation, using a Thermo Scientific Multiskan GO system (Thermo Fisher Scientific, Inc., Waltham, MA, USA). We developed a new software, VizELISA, for processing and visualization of glycan-array data (see Supplementary Material File S1 for details and manual).

### 4.6. Comprehensive Microarray Polymer Profiling

CoMPP analysis was performed on the leaf, stem and root tissues of the eggplant lines “305E40” and “67/3” (4 biological replicates each), as well as pooled samples of cultivar “Black Beauty” fruit skin and pulp (with each fruit sample representing a pooled sample of 5 biological replicates). Two sequential extractions (50 mM CDTA and 4 M NaOH) were performed on 10 mg per sample to obtain pectin-rich and hemicellulose-rich fractions, respectively. These extracts were probed with a selection of 32 mAbs and CBMs (Table 1) using the CoMPP method as described by Kračun et al. [26]. The resulting dataset was normalized globally, i.e., the highest mean signal obtained was assigned a value of 100 and all other data points were adjusted relatively across all probes and samples tested. A lower cut-off value of 5 was imposed.

## 5. Conclusions

In this work, we established an ELISA protocol for analyzing plant cell-wall (PCW) polysaccharides, which was applied to eggplant organs (fruits, leaves, stems and roots) along with our newly developed software, VizELISA, for processing and visualization of glycan-array data. Comprehensive microarray polymer profiling (CoMPP) was employed in addition to our ELISA method, testing with a variety of different probes; the results and data-processing strategies were compared directly. GC-MS was used to provide supportive data for the observations made on ELISA and CoMPP outcomes.

To conclude, our study provided a comprehensive characterization of the cell wall polysaccharides and glycoproteins in eggplant, covering three genotypes (“Black Beauty,” “305E40”, and “67/3”) and five tissue types (leaf, stem, root, fruit skin, and fruit flesh). We also confirmed the presence of a broad range of pectin, hemicellulose, AGP and extensin epitopes in eggplant tissues, many of which have not been studied before in eggplants, to our knowledge. Previous work on eggplant cell wall polysaccharides was mostly limited to fruit softening and how the activity of cell wall-modifying enzymes affects chilling injuries in eggplant fruits during cold storage. Our study is the most comprehensive analysis of eggplant cell walls published to date, with 32 total cell-wall polymer-related epitopes targeted. Our findings highlighted the patterns of relative abundance of these epitopes between different organs (leaves, stems, and roots) and different fruit tissues (exocarp compared to mesocarp, placenta and core tissues). The patterns of relative abundance between leaf, stem, and root tissues were largely consistent between the “305E40” and “67/3” lines.

## Figures and Tables

**Table 1:** Monoclonal antibodies and carbohydrate-binding modules used for CoMPP and ELISA.

Code	Epitope	Isotype	Reference
JIM5	Partially methyl-esterified homogalacturonan	IgG	[59,60]
JIM7	Methyl-esterified homogalacturonan	IgA	[59,60]
LM18	Partially methyl-esterified homogalacturonan	IgG	[61]
LM19	Unesterified homogalacturonan	IgM	[61,62,63]
LM20	Methyl-esterified homogalacturonan	IgM	[61,63]
LM7	Partially methyl-esterified homogalacturonan	IgM	[59,64]
RU2	Backbone of RG-1RG-1	IgM	[65]
RU1	Backbone of RG-1RG-1	IgG	[65]
LM5	Beta-1,4-galactan	IgG	[66]
LM6	Alpha-1,5-arabinan	IgG	[61,67]
LM16	Galactosylated RG-1RG-1	IgM	[61]
LM21	Beta-1,4-mannan	IgM	[62]
LM15	Xylosyl residues of xyloglucan	IgG2c	[53]
LM24	Galactosyl residues of xyloglucan	IgG2a	[52]
LM25	Xylosyl/galactosyl residues	IgM	[52]
LM11	Beta-1,4-xylan	IgM	[54]
LM23	Xylosyl residues	IgM	[52,68]
JIM8	Arabinogalactan-protein	IgG2c	[60,69,70]
JIM13	Arabinogalactan-protein	IgM	[60,69,70]
JIM15	Arabinogalactan-protein	IgM	[60,69,70]
JIM16	Galactosyl residue of AGP	IgM	[60,69,70]
LM2	Glucuronosyl residue of AGP	IgM	[71]
BS400-2	(1-3)-beta-D-glucan	IgG	[72]
BS400-3	(1-3; 1-4)-beta-glucans	IgG	[73]
CBM3a	Cellulose (crystalline)	CBM	[74]
LM8 *	Xylogalacturonan	IgM	[64]
LM13 *	Alpha-1,5-arabinan	IgM	[61,69]
JIM17 *	Arabinogalactan	IgM	[75]
MAC207 *	Arabinogalactan protein	IgM	[76]
LM1 *	Extensin glycoprotein	IgM	[77,78]
JIM11 *	Extensin glycoprotein	IgG2c	[77,78]
JIM20 *	Extensin glycoprotein	IgM	[77,78]

* Used exclusively with CoMPP.

## Data Availability

The original contributions presented in this study are included in the article/supplementary material. Further inquiries can be directed to the corresponding author.

## Supplementary Materials

The following supporting information can be downloaded at https://www.mdpi.com/article/10.3390/plants15203195/s1, VizELISA Software development for data processing and visualization of ELISA data. Figure S1. CoMPP (Comprehensive Microarray Polymer Profiling) applied to the analysis of tobacco leaves across three chemical fractions. CDTA, NaOH and cadoxen extractions were performed sequentially, after which the liquid extracts were tested for the relative abundance of plant cell wall-associated epitopes using monoclonal antibodies and carbohydrate binding modules. The values presented in the heatmap represent the mean spot values obtained from CoMPP, normalised to 100. I.e., the highest value across all probes was set to 100 and all other values were adjusted relative thereto. Values below 5 were removed. Figure S2. Different normalisation strategies applied to the visualisation of glycan microarray data. (a) Pre-normalised data is compared to examples of (b) global and (c) local normalisation. Global normalisation entails the normalisation of all samples to the highest signal obtained in the microarray, regardless of sample type or probe, where values below 5 are often omitted. Local normalisation entails normalising the values to the local maximum observed for each probe. In other words, each probe will have a value of 100 with all other samples normalised relatively thereto. Figure S3. Examples of heatmaps generated with VizELISA. The software allows for the separation of the generated heatmaps according to extractions, ideally for small sample sets (a). Large sample sets should ideally be presented in the style typical of CoMPP and glycome profiling (b). In order to gain insight into inter-sample variability and significant difference among groups, bar plots may be generated using smaller subsets of samples (Figure 5). Figure S4. A correlation matrix showing the correlation between leaf age and abundance of selected polysaccharide epitopes. Spearman correlation was used to give a correlation value between 1 and −1 for each pair of two probes and for both extractions. A value of 1 indicates a perfect positive correlation, whereas a value of −1 indicates a perfect negative correlation. Figure S5. Bar graph indicating the relative abundance of pectic polysaccharides in eggplant leaf, stem and root tissues. The presence and abundance of each polysaccharide in CDTA and NaOH are indicated with positive (white background) and negative (blue background) y-values values, respectively. Statistical significance between groups is indicated using letters; no significant differences were found between groups that share the same letter. The values for each polysaccharide were normalised relative to the maximum absorbance value obtained among the tested samples. Figure S6. A simplified standard operating procedure for processing and visualising data using VizELISA. The graphic user interface is divided into 4 sections: Data Input, Quick Parameters, Graph Specifications, and Standard Output. All output and feedback to the user is given via the Standard Output.

## Abbreviations

The following abbreviations are used in this manuscript:

AIR	Alcohol-insoluble residue
Ara	Arabinose
CoMPP	Comprehensive microarray polymer profiling
ELISA	Enzyme-linked immunosorbent assay
Fuc	Fucose
Gal	Galactose
GalA	Galacturonic acid
GC-MS	Gas chromatography–mass spectrometry
Glc	Glucose
GlcA	Glucuronic acid
GlcN	Glucosamine.
Man	Mannose
PCW	Plant cell wall
Rha	Rhamnose
Xyl	Xylose
