# Supplementary material for: Profiling the Main Cell Wall Polysaccharides of the Cultivated Eggplant (Solanum melongena L.) Using CoMPP and ELISA
Source: Plants (Basel). 2026 Oct 18;15(20):3195. doi: 10.3390/plants15203195 (PMC13709145; doi:10.3390/plants15203195)
Supplement: Supplementary file 1 [file plants-15-03195-s001.zip › plants-4632111-supplementary.pdf]

SUPPLEMENTARY MATERIAL

VizELISA Software development for data processing and visualisation of ELISA data

1. Data processing strategies for glycan array data

The common sources of variation and error rates in ELISA-based systems were identified and evaluated at least three decades ago (Watson & Musher, 1993). These researchers found significant between-technician variation. The volume of data produced by contemporary microarray systems for studying plant cell wall polymers (PCWPs) provides a significant challenge in terms of consistent data processing in addition to performing the physical assays. The number of software solutions dedicated to processing and visualising semi-quantitative ELISA or microarray data for PCWPs is limited. On the other hand, there are several tools dedicated to quantitative analysis of such data – some of which were developed nearly four decades ago (Butler et al., 1987). Quantification of target substances is important for most ELISA-based applications. In such a case, a standard curve is set up after testing a dilution series of the target compound in its purified form. These experiments rely on methods such as four-parameter or five-parameter logistic regression (4PL, 5PL) to create a model and quantify tested samples, and typically focus on a small number of target molecules (Avci, Pattathil, & Hahn, 2012; Danielak et al., 2020). However, this is rarely the case when using glycan microarrays to study plant cell wall polysaccharides and glycoproteins. This is because the diversity and complexity of cell wall polysaccharides, together with the sheer number of possible epitopes, provide considerable challenges when attempting to generate purified forms for each epitope (Pedersen et al., 2012). The epitopes for which we do have purified forms either need to be synthesised chemically or extracted from plant material, each of which strategies has its inherent advantages and disadvantages (Pattathil et al., 2015). Regardless, 4PL is commonly used to quantify substances using data obtained from an ELISA and a few online and offline solutions exist for this purpose (Akune et al., 2022; Butler et al., 1987; Danielak et al., 2020; GraphPad Software, 2023; MyAssays, 2009).

Most attempts at characterising the plant cell wall for a particular organism or tissue type rely on semi-qualitative data and do not acknowledge the inherent non-linear relationship between the concentration of a target polysaccharide and absorbance obtained by an ELISA, most often using heatmaps to visualise differences in antibody response between samples. The software solutions mentioned above all require significant manual input from the user. While these

programs are useful for routine ELISA tests using a small number of antibodies, there is a need for a software solution to simplify the task of processing and visualising large amounts of data from a plant cell wall characterisation endeavour. Researchers commonly use programs such as Microsoft Excel (Microsoft Corporation, 2023) or related software, which are often tedious to replicate for different experiments and leave room for human error. The Python3 programming language allows for the automation of data processing using packages such as Pandas and Scipi, and visualisation through heatmaps using packages such as Matplotlib or Seaborn (Bassi, 2007; McKinney Wes, 2010; Virtanen et al., 2020; Waskom, 2021). However, the lack of a defined graphic user interface and the required knowledge of programming using scripting languages poses a barrier to many. A guide for different statistical tests (random forest, principal component analysis, multiple factor analysis, etc.) and visualisation options using the R programming language has been provided for glycan microarray data (Nissen et al., 2023). The Python3-based program, CarbArrayART, is the only apparent software available specifically for handling carbohydrate microarray data, but lacks large-scale multi-file processing capacity (Akune et al., 2022). Additionally, the software allows for exporting of data in a format from which one can use another program to create heatmaps. Thus, CarbArrayART entails manual pre- and post-processing intervention from the user. To our knowledge, no other program exists for the purposes of processing and visualising (in the form of heatmaps) glycan microarray data specifically for cell wall profiling. Therefore, in order to reduce the amount of tedious manual intervention from researchers, there is a need for specialised software that will simplify the data analysis of cell wall profiling and the generation of informative figures such as heatmaps.

Heatmaps are among the most common modes of visualisation for glycan microarray data, since data can be condensed into an intuitive representation of a tissue type's polysaccharide profile. Along with the first description of CoMPP, Moller et al. (2007) recommended an online tool for the creation of heatmaps for cell wall profiling data (http://bbc.botany.utoronto.ca/ntools/cgi-bin/ntools_heatmapper.cgi) (I. Moller et al., 2007). This method of visualising cell wall profiling data through heatmaps often groups the major classes of plant cell wall polysaccharides for easy recognition of patterns between said groups. Figure S1 showcases this method by comparing the polysaccharide levels in tobacco leaves across different chemical fractions of the same cell wall material (Nguema-Ona et al., 2012).

	HG (mAb LM7)	HG (mAb JIM5)	HG (mAb LM18)	(1→4)-β-D-galactan (mAB LM5)	(1→5)-α-L-arabinan (mAB LM6)	(1→5)-α-L-arabinan (mAB LM13)	Xyloglucan (mAB LM15)	(1→4)-β-D-xylan (mAB LM10)	(1→4)-β-D-xylan/arabinoxylan (mAB LM11)	Feruloylated arabinan (mAb LM12)	(1→3)-β-D-glucan (mAb BS-400-4)	(1→4)-β-D-mannan (mAb BS-400-2)	(1→4)-β-D-mannan (mAb LM21)	Cellulose (CBM3a)	Cellulose (CBM4-1)	Cellulose (CBM9-2)	AGP (mAb LM2)	AGP (mAB JIM8)	AGP (mAb JIM13)	AGP (mAb JIM14)	Extensin (mAB LM1)	Extensin (mAb JIM19)
CDTA	88	35	44	18	26	0	0	0	0	0	0	0	0	0	0	0	0	17	21	0	10	0
NaOH	0	0	0	43	31	6	44	0	0	0	22	37	21	6	0	0	0	0	26	0	0	0
Cadoxen	0	0	5	9	0	0	14	0	10	0	8	13	18	44	9	0	0	0	0	0	0	0

HG, Homogalacturonan; AGP, Arabinogalactan protein

Scale 0 5 10 15 20 >25

Figure S1. CoMPP (Comprehensive Microarray Polymer Profiling) applied to the analysis of tobacco leaves across three chemical fractions. CDTA, NaOH and cadoxen extractions were performed sequentially, after which the liquid extracts were tested for the relative abundance of plant cell wall-associated epitopes using monoclonal antibodies and carbohydrate binding modules. The values presented in the heatmap represent the mean spot values obtained from CoMPP, normalised to 100. I.e., the highest value across all probes was set to 100 and all other values were adjusted relative thereto. Values below 5 were removed. Adapted from (Nguema-Ona et al., 2012).

This method of visualisation makes use of global normalisation, in which the absorbance values for all tested probes are assigned a value of 1 to 100 relative to the global maximum value. Although this approach is widely used, it created room for misinterpretation due to the non-linear nature of the enzyme-response relationship (Danielak et al., 2020; Herman et al., 2008; Nummer et al., 2018). When performing an ELISA, the binding of antibodies, and the data subsequently obtained, is inherently non-linear and relative values cannot be interpreted as relative amounts of the polysaccharides in question without first setting up calibration curves for each tested antibody-epitope pair. This is particularly difficult for plant cell wall polysaccharides due to the complex structures and branching patterns they exhibit. This often leads to antibodies being developed against a particular polysaccharide without the exact epitope being known. Moreover, antibodies used for glycan microarrays have inherently different avidities for their epitopes, which may introduce additional inaccuracies when normalising the data between different antibodies. Similar values on such a heatmap would thus represent different amounts of the respective epitopes present in the sample. A simple solution to this would be to set up an accurate calibration curve for each antibody using the relevant target PCWPs.

Here we propose an alternative approach to the global normalisation of glycan microarray data – normalisation of array data across each individual antibody. In other words, the maximum

absorbance value for each probe is used to relatively quantify only the values obtained for that probe (Figure S2). Although the problem relating to the varying binding avidities between antibodies is averted, the non-linear relationship between target abundance and absorbance still presents a challenge. However, data normalised using this strategy still gives the reader an intuitive understanding regarding the increase or decrease of a polysaccharide between samples, albeit a non-linear relationship. The same cannot be said for data between different antibodies. It would be inaccurate to assume a lower presence of a polysaccharide due to a lower absorbance reading when comparing different samples. Hence, each antibody should be evaluated separately.

As mentioned previously, it is impossible to determine the absolute quantification of a polysaccharide using microarray technologies alone due to the diversity of plant cell wall polysaccharides, coupled with the lack of purified standards. Additionally, the signals obtained from glycan microarrays are inherently non-linear with respect to the concentration of the targeted polysaccharide. Glycan microarray technologies provide a qualitative measure of the relative abundance of a cell wall component. However, non-linearity is seldom discussed in studies using these technologies to compare the cell wall profile of different samples. For instance, the interpretation of the most popular visualisation strategy for glycan microarrays, heatmaps, is often conducted with the assumption of direct proportionality. The manner in which heatmaps are normalised can emphasise the relative signals between certain probes over others (Figure S2). For a particular antibody, one might be tempted to interpret a value of 25 to indicate a polysaccharide concentration half of that when compared to a value of 50, however, non-linearity should be kept in mind. A theoretical solution to this problem would be to conduct a preliminary test of all samples for a particular antibody, selecting the sample with the highest signal and re-testing a dilution series of said sample alongside all other samples. Thereafter a calibration curve may be established on which the signals for all samples may be plotted and quantified relative to the sample with the highest concentration. Only then, after accounting for non-linear binding patterns associated with glycan microarrays, may one fairly compare the relative levels of cell wall polysaccharides or proteins between samples. Two different normalisation strategies are compared in Figure S2. It is important to note the null values that are assigned to samples when normalising across multiple probes (Figure S2 b). This is an example of higher signals drowning out signals two orders of magnitude lower, which can still provide useful information when normalising the values for each probe individually (Figure S2 c).

Even though valuable information can be generated regarding the structure and abundance of plant cell wall polysaccharides and glycoproteins using glycan arrays such as glycome profiling and CoMPP, limited deductions may be made using this information alone. Moller et al. (2007) posed CoMPP as a potential addition to larger systems-based approaches for studying plant cell wall components (Moller et al., 2007). Thus, these glycan microarrays should be employed as part of an arsenal of complementary methods (Chapter 2).

a)

	Probe 1	Probe 2	Probe 3
Sample 1	50	10	0,2
Sample 2	32	9	0,3
Sample 3	21	1	0,9
Sample 4	15	1,5	0,55

b)

	Probe 1	Probe 2	Probe 3
Sample 1	100	20	0
Sample 2	64	18	0
Sample 3	42	0	0
Sample 4	30	0	0

c)

	Probe 1	Probe 2	Probe 3
Sample 1	100	100	22
Sample 2	64	90	33
Sample 3	42	10	100
Sample 4	30	15	61

Figure S2. Different normalisation strategies applied to the visualisation of glycan microarray data. (**a**) Pre-normalised data is compared to examples of (**b**) global and (**c**) local normalisation. Global normalisation entails the normalisation of all samples to the highest signal obtained in the microarray, regardless of sample type or probe, where values below 5 are often omitted. Local normalisation entails normalising the values to the local maximum observed for each probe. In other words, each probe will have a value of 100 with all other samples normalised relatively thereto.

2. Data input

During this study, a dedicated software tool, Visualisation tool for Enzyme-Linked Immunosorbent Assay (VizELISA), was created to reduce the manual labour often required to process and visualise large amounts of glycan array data. The software was tailored to ELISA analyses for characterising plant cell wall glycans. The data is given to VizELISA in the form of Microsoft Excel spreadsheets (.xlsx) containing raw glycan array data and heatmaps are generated therefrom in the style commonly used for cell wall polymer characterisation (Avci, Pattathil, Miller, et al., 2012; Kračun et al., 2017).

2.1. Raw data input

Data input for VizELISA is automated and requires minimal user intervention. Manual intervention is limited to where users are required to follow a particular naming scheme for samples and data files; all other input will be given via the VizELISA Graphical User Interface (GUI). Raw data files (each containing the absorbance values for a single plate) should be

named in accordance with the abbreviated code of the antibody used for the analysis of the respective plate. VizELISA is set up to automatically populate generated figures using the full epitope description associated with each antibody code, obtained from a database integrated with VizELISA. In the case where an antibody code is not recognised, a dedicated error code will guide the user through the simple process of manually updating the database with information regarding the antibody in question. Information regarding the antibodies used during a particular analysis as well as the samples analysed will be summarised and included in the output file for said analysis.

A detailed mapping file, or template, is provided (in the VizELISA installation folder) in the form of a Microsoft Excel spreadsheet (.xlsx) in which the user can visually map sample locations on one or more 96-well plates. Identical sample codes on a 96-well template are interpreted as technical replicates and are automatically averaged with the standard deviation included in the report file after the execution of VizELISA. The user can also choose to use the median of technical replicates as the representative value for each sample on the included template. It is also on this template where the user can indicate the order in which the samples will be processed or restrict the downstream analyses to a subset of the samples indicated on the template. Ten 96-well templates can be found as separate sheets on the included template file and more can be added by the user by simply duplicating and renaming one of the other sheets. In the case where samples are spread over several plates, the corresponding number of templates are used, each found on a separate sheet within the template file. Raw data files (each containing the absorbance values for a single plate) should be named in such a way that the suffix of the file name corresponds to the relevant sheet number in the included template file. For example, if samples are mapped over 3 plates (i.e., 3 template sheets), the output for each analysis should be named Antibody_1, Antibody_2, and Antibody_3. Sample mapping on the template file should ideally be completed before analysis during the experimental planning phase. Thus, the user will be aware of the data file naming scheme that will be required for data processing and can rename each file appropriately during the data generation phase. This aids record keeping and potentially also saves time spent on renaming numerous raw data files manually.

As is often the case with ELISA experiments analysing PCWPs, multiple sequential extractions are often performed that extract these polymers based on how closely they are associated with the cell wall matrix. For example, hot water or CDTA (a chelating agent) are used to solubilise pectic polysaccharides, whereas varying degrees of alkaline extractions target more closely

associated polymers. In cases where multiple extractions are used, sample names are given suffixes that correspond to the respective extractions performed. For example, "CDTA_sample1" and "NaOH_sample1" correspond to different extractions performed on the same sample and the resulting data will be processed as such.

2.2. Input data validation

An optional button can be found via the VizELISA GUI that allows users to check input data prior to data processing. This functionality checks whether data files are in the correct order, whether extraction labels are present (if relevant), whether the provided raw data files are in an acceptable format and whether the data files contain only integers and floating-point values (except for dedicated string codes allocated to outliers). An extensive library of error codes provides users with guidance and solutions if the provided input data files are not correct (see Error Handling).

3. Data processing

3.1. Outlier removal

VizELISA accounts for both manual and automatic removal of outliers, and a detailed overview of outlier removal is included in the output report file. The user can indicate whether outliers in technical replicates should be removed automatically, granted that at least three technical replicates are present. Alternatively, the user may assign a code dedicated to technical outliers (via the VizELISA GUI) and replace the outlying raw data values with said code. Users may also opt for the automatic removal of biological outliers, e.g., for the generation of bar plots, however, users are urged to monitor the resulting report file in which all data manipulations and outlier removal are documented. Alternatively, two manual approaches can be taken to remove entire samples from downstream analyses. The first approach is to exclude the outlying samples when indicating sample/sample subset order, however, this will exclude this sample for all probes tested. The second method entails replacing all technical replicate values with the above-mentioned outlier code. This will exclude the sample in question for the analysis of a single antibody only.

3.2. Statistics

Depending on the nature of the samples tested, users can indicate optionally whether the analysed data are serial or independent, an option that dictates the downstream statistical tests that will be performed. Serial data includes concentration series or data points that are linked temporally (e.g., data points representing an increasing series of ELISA incubation times). In

addition to selecting “serial data”, users may also indicate whether the data points represent continuous or ordinal data, which in turn determines the statistical correlation to calculate Pearson or Spearman correlation, respectively. Similarly, users can also indicate to calculate an R-squared value if relevant to their analysis. While heatmaps are the main focus of VizELISA, the software also includes the functionality of generating correlation matrices, line graphs, scatter plots and bar plots from the entire analysed dataset or on a user-provided restricted subset of the analysed data. Statistical tests are performed where relevant, including a Two-Sample *t*-test and Tukey’s method. In the case of bar plots, the resulting statistical significance can be indicated on the bar plots. Users may indicate custom symbols for different P-value thresholds, however, by default a double asterisk is used for P-values below 0.01 and a single asterisk is used for P-values between 0.01 and 0.05.

As mentioned under Data Input, identical sample codes in the included 96-well plate mapping file will be interpreted as technical replicates for the respective sample. Users have the option to use the median for the technical replicates of each sample via the VizELISA GUI. Alternatively, the mean of technical replicates will be used by default.

3.3. Error handling

VizELISA has an extensive collection of error messages that will be displayed when the corresponding errors have been encountered. These messages provide feedback and possible solutions to encountered errors and exist to familiarise users with VizELISA and streamline future analyses. In the event of an error occurring during an analysis, VizELISA will be terminated, and a relevant error message will be presented under Standard Output. The relevant error message will also be included in the report file for said analysis for record-keeping and to aid future troubleshooting efforts.

3.4. Report generation

VizELISA generates a report after performing each analysis with a comprehensive collection of user settings, descriptive statistics, data processing that was performed, and reference codes for any errors that may have been encountered during the analysis. User settings for each analysis will be saved and included at the top of each report file for easy reference. This is to promote reproducibility between analyses, even between different users. To promote reproducibility and transparency further, each data processing step is summarised and

displayed as a continuous feed in the Standard Output section of the VizELISA GUI. Information such as outlier removal (both manual and automatic for both technical and biological replicates) will be also displayed in this section.

In the VizELISA folder, a compressed folder will be generated for each analysis under Output containing the following:

- Report file including time stamps
- All generated graphs (as PNG files)
- Microsoft Excel spreadsheet containing all processed data in the form of a table.

4. Visualisation

4.1. Heatmaps

Heatmaps are the main output of VizELISA. VizELISA differs from other heatmap tools in two key ways. The first distinction lies in its automated file handling which is not found in other heatmapping tools, coupled with data processing capabilities where hundreds of raw data files can be read, processed, and displayed on a custom heatmap in seconds. Using user-provided instructions, VizELISA extracts the relevant data from multiple raw data files and stores it in the form of a Pandas dataframe after relevant data processing and normalisation have been performed. This dataframe will be used to create a heatmap as per user instructions but will also be exported as a Microsoft Excel spreadsheet in case other software is preferred for heatmap generation. The second key difference between VizELISA and other heatmapping tools lies in its approach to the normalisation of data. CoMPP analyses are typically visualised on heatmaps that are normalised globally (i.e., the highest signal obtained between all probes and all samples is set to 100 and all other values are transformed in proportion thereto). This mode of data normalisation (Figure S2) can be chosen via the VizELISA GUI, however, the software by default normalises data locally (i.e., the max value for each probe is set to 100 and all other samples are set relatively thereto). In other words, each column (representing a particular probe) will have a value of 100. Users have the option to include or remove numbers on the generated heatmap to better suit quantitative (Figure S3 a) or qualitative/semiquantitative data (Figure S3), respectively. Additionally, depending on whether the nature of the input samples allows for correlation testing, users may generate correlation matrices based on the correlation coefficient calculated (Figure S4). A heatmap will be generated to indicate visually any potential correlations between the tested polysaccharides. This graphical representation will indicate using colour whether these correlations are positive or negative. Antibodies are removed automatically if there is no numerical variation between samples for said antibodies.

(a)

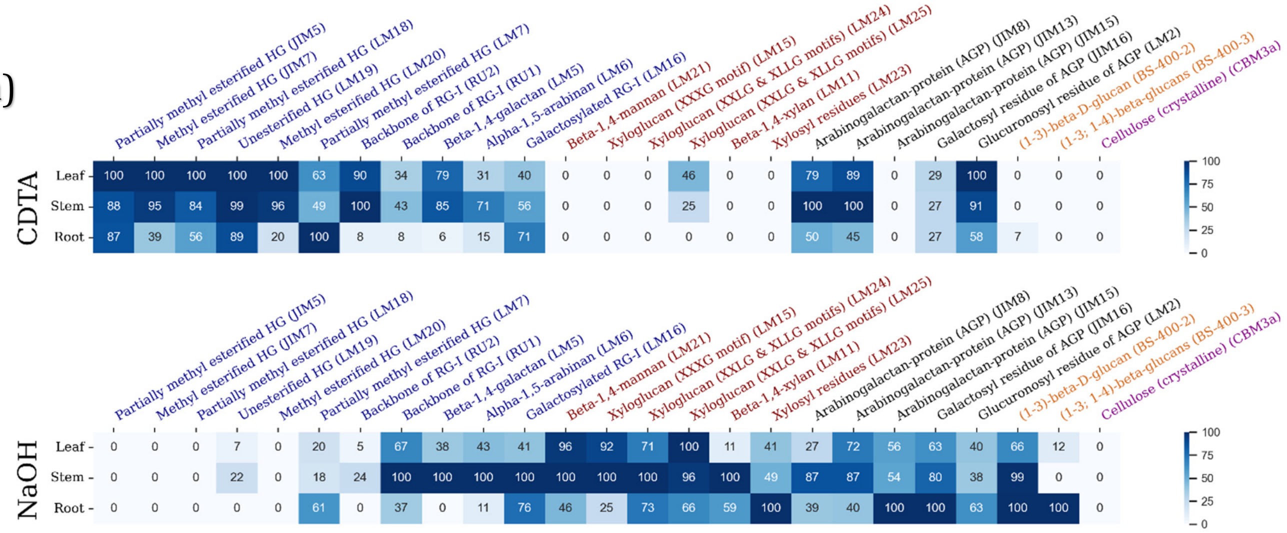

(b)

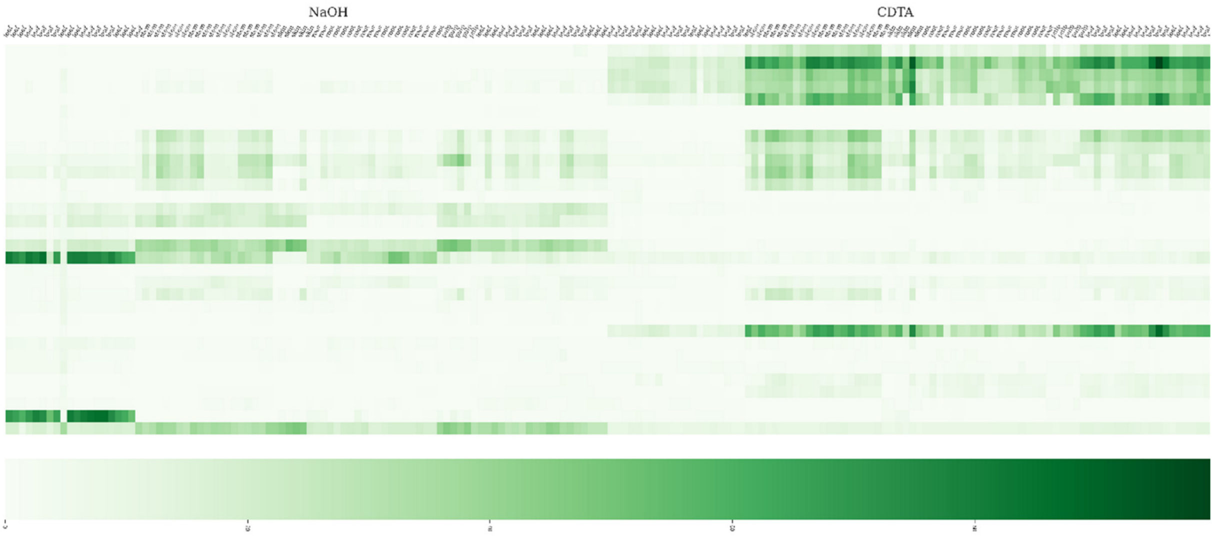

Figure S3. Examples of heatmaps generated with VizELISA. The software allows for the separation of the generated heatmaps according to extractions, ideally for small sample sets (a). Large sample sets should ideally be presented in the style typical of CoMPP and glycome profiling (b). In order to gain insight into inter-sample variability and significant difference among groups, bar plots may be generated using smaller subsets of samples (**Figure S5**).

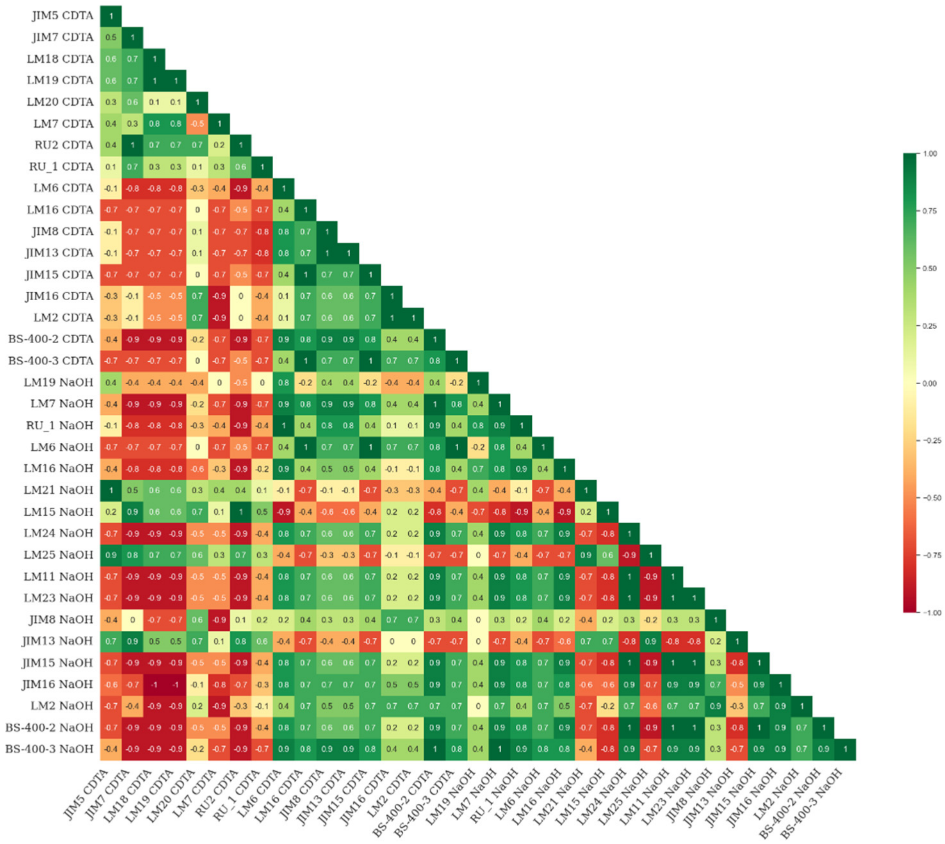

Figure S4. A correlation matrix showing the correlation between leaf age and abundance of selected polysaccharide epitopes. Spearman correlation was used to give a correlation value between 1 and -1 for each pair of two probes and for both extractions. A value of 1 indicates a perfect positive correlation, whereas a value of -1 indicates a perfect negative correlation.

4.2. Bar plots

Users can indicate between one and four groups of samples to be plotted on bar graphs (Figure S5). Depending on the number of groups and the nature of their data, significance testing can be done and visualised in order to indicate statistical differences between samples. By default, differences with P-values below 0.01 are indicated with a double asterisk (**) and P-values between 0.01 and 0.05 are indicated with a single asterisk (*). However, this mode of significance indication is only applicable when two groups or two sets of two groups are being compared, e.g., leaf mutant vs leaf control paired with root mutant vs root control. Users have the option to indicate custom symbols and P-value thresholds.

By default, VizELISA groups polymers into three major classes: pectins, AGPs, and cellulose and hemicellulose. Custom groupings of probes can be supplied by the user under the Data Input section of the VizELISA GUI. Users can choose whether different cell wall extractions (for example, CDTA, NaOH, etc.) should be visualised on the same plot (only if two extractions were used) or on separate plots.

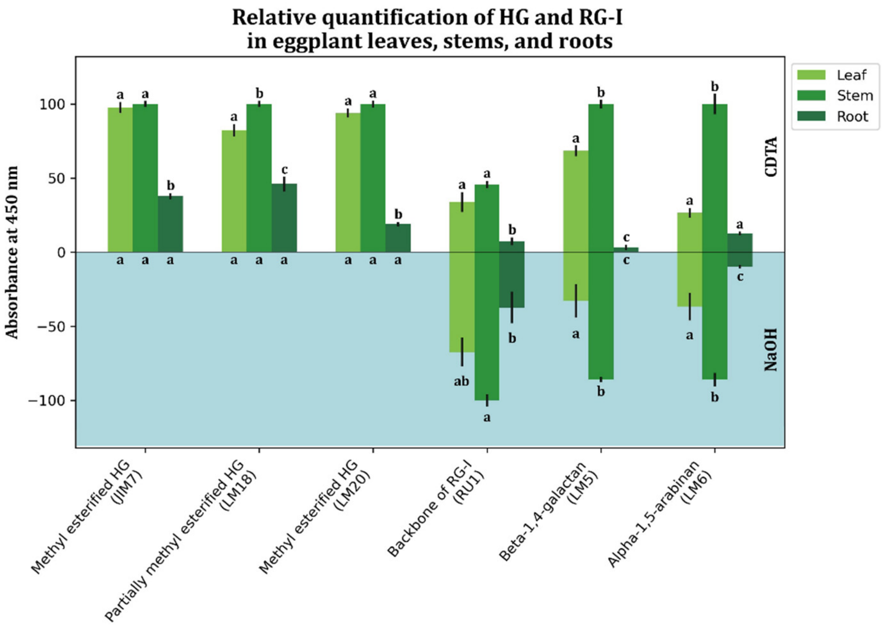

Figure S5. Bar graph indicating the relative abundance of pectic polysaccharides in eggplant leaf, stem and root tissues. The presence and abundance of each polysaccharide in CDTA and NaOH are indicated with positive (white background) and negative (blue background) y-values values, respectively. Statistical significance between groups is indicated using letters; no significant differences were found between groups that share the same letter. The values for each polysaccharide were normalised relative to the maximum absorbance value obtained among the tested samples.

5. User interface

The simple user interface was built using Customtkinter, a Python library based on Tkinter. The GUI follows a minimal design pattern and is broken up into four main sections: Data Input, Quick Parameters, Graph Specifications, and Standard Output (Figure S6). Data Input allows users to open the data mapping file and to indicate characteristics of their input data that are required for the bulk processing of their data files. There are also options for renaming files in bulk and checking whether the input files are correctly named and formatted for further processing. Lastly, it is under this section where users indicate which antibodies (with their respective abbreviated codes) are included in the analysis. Quick Parameters contain mostly checkboxes for which users indicate technical preferences regarding data processing, e.g., whether or not all antibodies will be normalised globally or individually, etc. The Graph Specifications section houses settings that are related to the visual outputs of VizELISA and several text fields can be found here in which users indicate preferred axis titles, as well as a number of other parameters that can be changed such as font specifications, colour schemes, etc. The Standard Output section houses a window to which Python’s standard output has been redirected. Here a stream of information will inform users about the progress in terms of data

input steps, data processing steps and, finally, visualisation. Any errors encountered will be displayed here as well as an error description to aid in troubleshooting. Important preferences and settings given by users via the VizELISA GUI, as well as any errors encountered, will be included in the generated report file for reproducibility and troubleshooting.

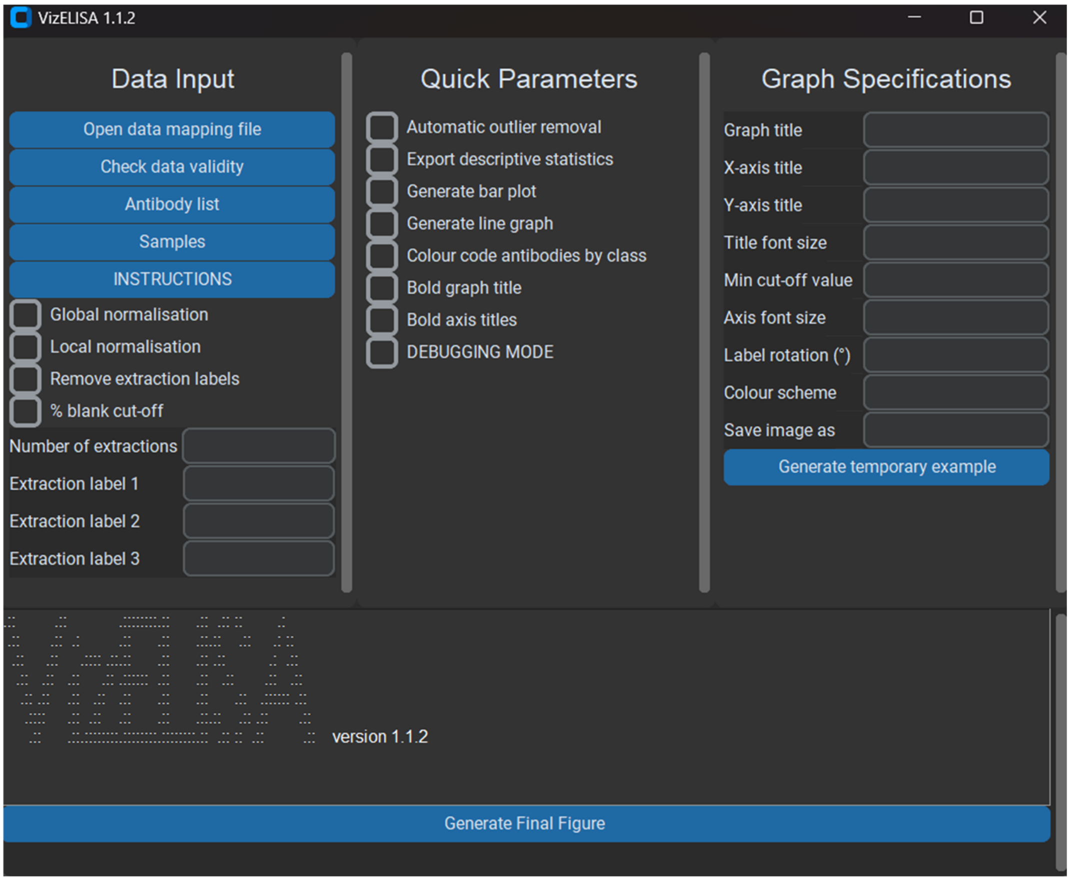

Figure S6. A simplified standard operating procedure for processing and visualising data using VizELISA. The graphic user interface is divided into 4 sections: Data Input, Quick Parameters, Graph Specifications, and Standard Output. All output and feedback to the user is given via the Standard Output.

6. Software availability

VizELISA is fully open source and available for download from the online code repository, Github, using the following URL:

https://github.com/egbaden/VizELISA.git
